# Exploring the applicability of “One-Size-Fits-All” road transport decarbonization strategies: a participatory energy systems modeling comparison of urban and non-urban municipalities

**DOI:** 10.1038/s41598-025-94579-w

**Published:** 2025-03-28

**Authors:** Maria de Oliveira Laurin, Vahid Aryanpur, Hadi Farabi-Asl, Maria Grahn, Maria Taljegard, Karl Vilén

**Affiliations:** 1 Department of Mechanics and Maritime Sciences, Chalmers University of Technology, 412 96 Gothenburg, Sweden; 2 University College Cork, SFI MaREI Centre for Energy, Climate and Marine, Environmental Research Institute, Cork, Ireland; 3 School of Engineering and Architecture, University College Cork, Cork, Ireland; 4 Division Built Environment, System Transition, RISE Research Institutes of Sweden, 412 58 Gothenburg, Sweden; 5 Department of Space, Earth, and Environment, Chalmers University of Technology, 412 96 Gothenburg, Sweden; 6 IVL Swedish Environmental Research Institute, 400 14, Gothenburg, Sweden

**Keywords:** Fossil-free road transport, Light-duty vehicles, Local energy systems, Medium and heavy-duty vehicles, Participatory modeling, Socio-geographical contexts, Environmental impact, Climate-change mitigation

## Abstract

Despite the key role that local authorities play in shaping energy policies and implementing action plans, their level of involvement has been insufficiently examined. This study aims to assess how different socio-geographical factors impact the adoption of fossil-free vehicle technologies and fuels for private cars, buses, and trucks. Using a participatory energy systems modeling approach, this study explores the cost-optimal decarbonization of road transport in four urban and non-urban Swedish municipalities. By collaborating with local authorities, socio-technical scenarios are modeled to reflect climate actions, resources and infrastructure availability, as well as travel patterns. The findings reveal a preference for lower upfront costs in urban areas with shorter trip distances, leading to a higher small-size battery electric vehicles (BEVs) share. Conversely, in non-urban areas with longer trip distances, fuel economy, fuel cost, as well as operation and maintenance costs outweigh upfront costs, increasing average-size BEVs share. Buses and trucks also experience a growing BEVs and fuel cell vehicles (FCEVs) share, driven by their typically high annual mileage. Biofuels play an intermediate role until BEVs and FCEVs are reduced in cost. Tailoring decarbonization strategies to local contexts is essential for maximum effectiveness, balancing national and regional climate goals with urban and non-urban challenges.

## Introduction

In light of the Paris Agreement^1^, the European Commission further promised to meet a net-zero emissions target by 2050^2^. Albeit this target, the European transport sector’s domestic greenhouse gas (GHG) emissions increased by 20% between 1990 and 2019^3^, highlighting the challenge associated with decarbonizing this energy sector. Within this challenge, a special focus falls on road transport as the major contributor to the total European transport-related emissions, with, in 2019, a total share of 69%^4^. Road transport’s (i) current fossil fuel reliance^5^; (ii) rooted technological lock-ins (e.g. Society car dependency)^6^; (iii) historical links to economic growth^7^; and, therefore; (iv) continuously increasing demand; persist in challenging the endeavor of decarbonizing this energy sector^8^. Overcoming the hurdles associated with road transport decarbonization, same as energy transitions in other sectors, calls for a comprehensive and holistic picture of the entire system. Meeting such a requirement is argued to benefit from the application of Energy Systems Modeling (ESM) tools^9,10^.

ESM is a well-established framework capable of assessing how a given real-world energy system might evolve under different stimuli, such as actions towards climate goals^11^. Such evaluation can integrate both a transition perspective (i.e. indications on how the system may need to develop towards meeting a given goal?) and a long-term understanding (i.e. what future energy mix will be in place when a given goal is met?), providing insights for the decision-makers in the energy transition^12^. Within ESM’s family, energy systems optimization models (ESOMs) are known to excel in iteratively interacting techno-economic perspectives, representing the whole energy system^11,13^. Unlike simulation models (i.e., predictive tools generating results based on historic data [see e.g. Ref.^14^]), optimization models explore the energy systems dynamic, through “if–then” or “what-if” questions over a specified long-term time horizon^11,15^. Some studies already use ESOMs to investigate the decarbonization of road transport (see, e.g. Ref.^16–19^). Yet, these studies tend to limit their understanding to an aggregated and conceptual perspective, using average values to define the techno-economic interactions of road transport decarbonization at continental^16^, national^17^, regional^18^, and large city^19^ levels. Spatial dynamics (i.e. commonly used as referring to how energy patterns – supply and demand –, infrastructure availability, and environmental factors interact and evolve across different geographical areas) are often overlooked in current literature^20^, dismissing that road transport decarbonization depends on socio-geographic conditions, e.g., travel patterns (such as car driving distances, and traveling routes), vehicles’ size and propulsion technologies, fuel consumption profiles, and traffic regulations (e.g. parking fees and maximum speed)^21–26^ that are local context-specific^27^.

Effectively addressing the decarbonization of road transport, like any energy transition, should not be restricted to a conceptual perspective (i.e. energy transitions should not be limited to a theoretical understanding), demanding to be complemented and adjusted to a local energy systems perspective^28^. Such a perspective portrays a social dimension related to energy transition^29,30^ by depicting the interactions and feedback loops between demand-side behavior and energy transitions^31,32^. Such a social dimension advocates public engagement, benefiting from a participatory approach (PA)^33^. By bringing together various local stakeholders and expertise, a PA integrates bottom-up local-specific knowledge of techno-economic and socio-geographic aspects. These aspects provide valuable insights not only into the operation dynamics of local communities and services^34–36^ but also their commitment to addressing climate issues and the challenges they face in this regard^37^. Assuming a PA can strengthen the social accessibility of energy transitions^38^, and therefore fostering local climate resilience^39^. Accordingly, adopting a PA becomes important to consider in any type of energy transition^40–43^. Some authors expand this thought to the ESM field by elaborating on the advantages of adopting a PA^44^ as (i) an effective way of linking theoretical objectives to practical and achievable goals^45^; (ii) enhancing the credibility and transparency of modeling results^46^; and (iii) fostering legitimate decision-making, as a result of balancing and comparing both the positive and negative feedbacks of the different possible outcomes, in a holistic way^47^.

Some literature on road transport already reviews the importance of merging ESM and PA^6,48–52^. The different authors use PA as a stakeholders’ narrative storytelling tool for both designing and evaluating the feasibility modeling scenarios. Similarly, the authors claim that modeling scenarios developed in a participatory manner effectively integrate a spatial dynamic dimension into the ESM exercises. Within the ESM field, two studies were found to combine ESOM and a PA in the context of road transport decarbonization^6,52^. Venturini et al.^6^ link a PA, as gathering stakeholders’ narratives on future road transport key driving forces, with ESOM, in the country-context of Denmark. The authors transcribe those narratives into quantitative modeling scenarios and assumptions as adding a spatial dynamic dimension to the ESOM exercise. As the main intake, the authors highlight that an ESOM, as a stand-alone exercise, performs a long-term techno-economical optimization, overlooking relevant behavioral, socio-political, and economic barriers – factors that can be included if assuming a PA during the modeling exercise. Fortes et al.^52^ compare the energy supply portfolio when decarbonizing different economic sectors, including road transport, using either a PA or ESOM at the country-level of Portugal. Despite visioning similarities, the authors present that (i) purely qualitative scenarios, as a direct outcome of a PA, fail in capturing long-term uncertainties, meaning that these scenarios might be neither cost-effective nor technically feasible, posing a challenge when considering a quantitative target; and (ii) ESOM tools dismiss that some decarbonizing road transport strategies are not aligned with the current socio-political and economic context. Appropriately, the authors suggest that energy transition understanding can benefit from applying a combined approach that establishes a complementary loop between a PA and ESOM exercise.

Two research gaps can be identified in the above-described studies. First, typical ESOM studies on road transport decarbonization have difficulties in capturing the challenges that local energy systems may face. Such an approach dismisses road transport decarbonization as being context-dependent. Further, as a second literature gap, no study was found to either (i) use a PA as a provider of a local energy system perspective; or (ii) integrate socio-technical scenarios developed in a participatory manner in ESOM that aim to investigate the role of road transport decarbonization in different types of municipalities and corresponding socio-geographical contexts.

Recognizing the abovementioned literature gaps associated with road transport decarbonization, this research provides a novel contribution to this scholarly discourse by developing an ESOM that focuses on a local energy systems perspective assessing scenarios based on participatory dynamics. The PA added to this ESOM is adopted from de Oliveira Laurin et al.^53^, where municipal officials contributed to developing decarbonization pathways. These pathways, as descriptive of different socio-geographical contexts (i.e. urban and non-urban municipalities), are exogenously incorporated in the modeling exercise, as modeling scenarios. Such a method is thus specifically tailored to serve the unique context of different Swedish municipalities at the local level. By doing so, a cost-optimization model is developed to quantitatively assess local socio-technical scenarios. These local scenarios, as a direct outcome from municipal officials PA, are modeled as testing different possible local futures, providing a comprehensive perspective of road transport decarbonization. Similarly, tailoring the model development to the specific context of local scenarios enriches the modeling spatial dynamics to focus on a local energy perspective. Due to the local aspect of the modeled scenarios, their outcome might vary according to different socio-geographical contexts. Therefore, the present study expands its aim to further explore if and how the socio-geographical context may influence the scenarios towards road transport decarbonization. The main questions to be answered are the following: RQ1. How to integrate a local spatial dynamic dimension in Energy Systems Optimization Models?; RQ2. How does the socio-geographical context influence road transport decarbonization, especially in terms of fleet and fuel mix for passenger cars, buses, and freight transport?

While this paper primarily delves into a Swedish case study, it provides a universal lesson on how to promote more sustainable and inclusive transport systems, offering applicable and valuable insights for an international audience and diverse stakeholders. Specifically, the findings address various dimensions of sustainable development goals (SDGs), notably SDGs 7, 11, and 13, making them relevant to national policy-making worldwide. By emphasizing the importance of a local perspective, this study advocates for nuanced policy interventions that account for the diverse socio-geographical factors influencing transport systems.

## Results

This section presents the outcomes from the TIMES model specifically developed according to the socio-geographical context of four Swedish municipalities, reflecting the participatory interaction of local stakeholders.

The results differed between urban and non-urban settings, yet similar trends were found in the different non-urban municipalities. Hence the results related to light-duty vehicles (i.e., passenger cars) are presented as depicting the most extreme differences between the considered urban municipality and the resulting average of the three non-urban municipalities.

By including assessments of future fleet and energy carriers (i.e. in this study defined as fuels) mix for buses as well as medium (i.e. weight < 3.5 tons) and heavy-duty trucks (i.e. weight ≥ 3.5 tons), model results can provide road transport cross-sectoral dynamics (i.e. identification of possible mobility synergies and fuel competition within the different categories of road transport) the corresponding results are aggregated. Separated results for each of these three transport segments are presented in Supplementary Information (see “S2. Additional Results*”*).

### Fleet mix

**Fig. 1:**
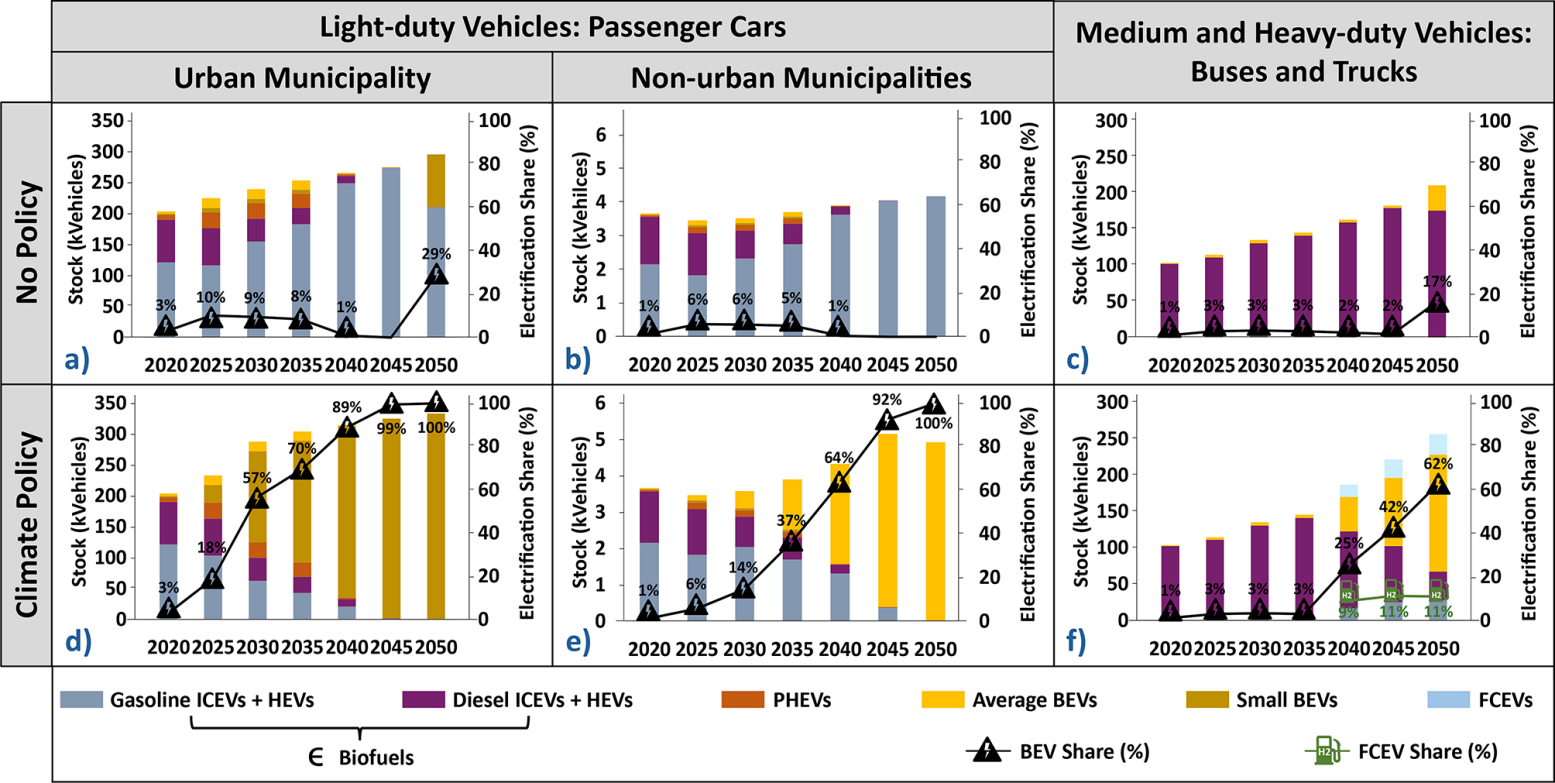
Fleet mix in the No-policy and Climate Policy Scenarios, for different road transport vehicle categories, presented in stock of thousand (k) vehicles (left axes). The black and green curves show the direct (i.e., BEVs) and indirect (FCEVs) electrification share (right axes), respectively. Fleet mix and electrification share in No Policy Scenario: (**a**) urban municipality; (**b**) non-urban municipalities; and (**c**) buses and medium and heavy-duty trucks. Fleet mix and electrification share in Climate Policy Scenario: (**d**) urban municipality; (**e**) non-urban municipalities; and (**f**) buses and medium and heavy-duty trucks. Biofuels – biogas and ethanol – are considered in the “Gasoline ICEVs + HEVs” category, while biodiesel (HVO100) is considered in “Diesel ICEVs + HEVs”. Detailed information about model constraints, parameters, and related assumptions as well as the separated results for each transport segment are further described in Supplementary Information (see “S1.4.Input Data and Modeling Assumptions” and “S2. Additional Results” Sections). BEVs, battery electric vehicles; FCEVs, fuel cell electric vehicles; ﻿ICEVs, internal combustion engine vehicles; HEVs, hybrid electricity vehicles; PHEVs, plug-in hybrid vehicles.

The *No Policy Scenario* (Fig. 1a–c) dismisses climate goals and related policies, defining the gradual increase in transport demand as the primary factor driving fleet dynamics change. For passenger cars, fleet electrification remains marginal in both urban (Fig. 1a) and non-urban municipalities (Fig. 1b), only represented by the existing fleet based on real data from 2020 to 2022. The least-cost solution for passenger cars is dominated by gasoline internal combustion engine vehicles (ICEVs). While non-urban municipalities maintain this trend, urban municipalities experience a shift towards small battery electric vehicles (BEVs) post-2045, driven by cost parity between small BEVs and gasoline ICEVs. Buses, medium, and heavy-duty trucks, with long annual mileage, prioritize fuel economy, remaining reliant on diesel ICEVs. However, due to technological developments and consequently upfront cost decreases over the years, both buses and medium trucks, due to shorter annual mileage comparing to heavy trucks, slowly shift towards a direct electrification preference. By 2030, BEVs represent 100% of the new bus fleet, with BEVs’ upfront cost being 10% higher than diesel ICEVs. Post-2045, BEVs represent 15% of the medium trucks fleet.

The *Climate Policy Scenario* (Fig. 1d–f) considers ambitious climate goals (i.e. light-duty vehicle carbon dioxide [CO_2_] emissions by 80% compared to 2010 by 2030^54^ and achieving carbon neutrality by 2045^55^) and road transport-related policies. The analysis shows a rapid acceleration in fleet electrification, particularly in urban municipalities (Fig. 1d), where, already by 2030, small BEVs compose more than 50% of the fleet. In non-urban municipalities (Fig. 1e), electrification becomes dominant post-2035, with the fleet composed mainly of average-size BEVs. This difference is due to varying average annual mileage (i.e. 10,400 km for urban and 12,200 km for non-urban municipalities) and average trip distances (i.e. 93% and 75% of trips are shorter or equal to 100 km for urban and non-urban municipalities, respectively), which influence vehicle choice. Small-size BEVs are preferred in urban areas due to their low upfront costs, while average-size BEVs, due to their lower total driving cost, are favored in non-urban areas. An average-size BEV, in comparison to a small-size BEV, is assumed to have a lower need for fast charging due to its larger battery capacity, which can support longer trip distances. However, this assumption may vary based on the specific travel patterns, as average BEVs still require intra-day charging for extended trips, factors exogenously considered in the modeling exercise. In response to the climate goals and related policies, both buses and medium-duty trucks increasingly adopt BEVs, while heavy-duty trucks shift to Fuel Cell Electric Vehicles (FCEVs) post-2040 due to their better performance (i.e. cargo capacity and longer average annual mileages) for long-haul trucks.

Overall, the shift in fleet dynamics aligns with the model’s cost-optimization logic, which prioritizes either low upfront costs or more efficient technologies, based on different travel patterns. It is worth noting that, from a cost-optimization perspective and in the long-term horizon, the model selection prioritizes both direct (in the case of passenger cars, buses, and medium-duty trucks) and indirect (in the case of heavy-duty trucks) electrification. This trend is driven by continuous technological advancements, further expanding the still niche markets for these alternative electrified road transport solutions. The increasing direct electrification aligns with historical and current trends, as evidenced by the annual rise in new BEVs registrations across all road transport segments^56^.

### Fuel mix and CO_2_ emissions

**Fig. 2:**
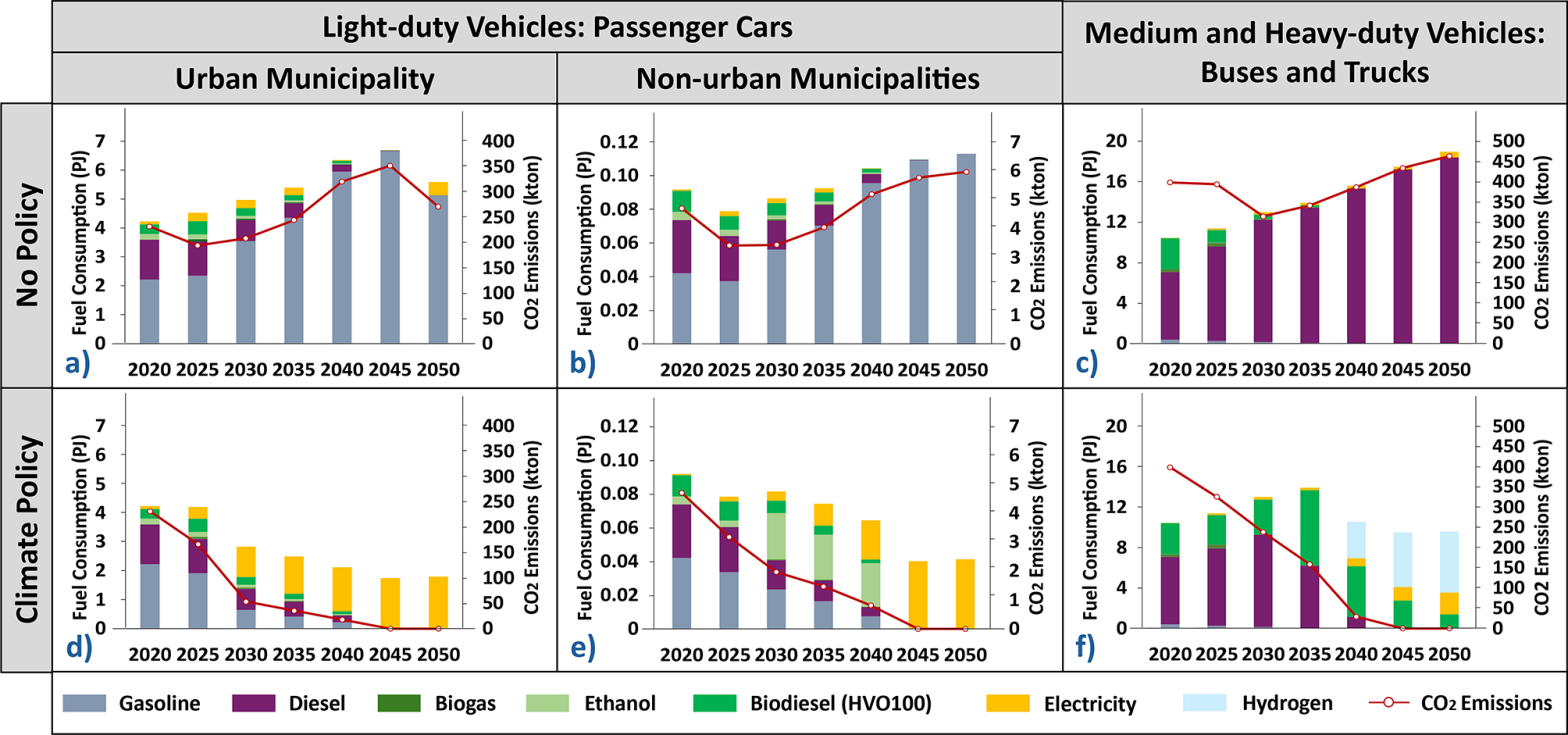
Fuel consumption (left axes) and resulting CO_2_ emissions (right axes) in the No Policy and Climate Policy Scenarios for different road transport vehicles categories. Fuel consumption and CO_2_ emissions in No Policy Scenario: (**a**) urban municipality; (**b**) non-urban municipalities; and (**c**) buses and medium and heavy-duty trucks. Fuel consumption and CO_2_ emissions in Climate Policy Scenario: (**d**) urban municipality; (**e**) non-urban municipalities; and (**f**) buses and medium and heavy-duty trucks. Detailed information about model constraints, parameters, and related assumptions as well as the separated results for each transport segment are further described in Supplementary Information (see “S1.4.Input Data and Modeling Assumptions” Section). The ethanol considered for light-duty vehicles is E85 and for buses, as well as medium and heavy-duty trucks ED95. CO_2_, carbon dioxide; HVO, hydrotreated vegetable oil.

In the *No Policy Scenario* (Fig. 2a–c), heavy reliance on light-duty vehicles on conventional ICEV powered by gasoline, coupled with an increasing transport demand, results in high fuel consumption in both urban (Fig. 2a) and non-urban municipalities (Fig. 2b). Due to gasoline dominance, passenger cars resulting CO_2_ emissions gradually increase. Notably, the decrease in CO_2_ emissions in both the urban and non-urban municipalities in 2025 is attributed to the existing modeled fleet calibrated using real data on new passenger car registrations from 2020 to 2022. In the urban municipality, the long-term decrease in CO_2_ emissions, experienced post-2045, is marked by an increasing adoption of small BEVs. Similarly, for buses, as well as medium and heavy-duty trucks (Fig. 2c), fuel consumption and resulting CO_2_ emissions, mirror their fleet mix heavily reliant on fossil diesel. Still, post-2030 for buses and post-2045 for medium trucks, BEVs are preferred, increasing the electricity consumption, which marginally decreases the emissions by 2050.

In the *Climate Policy Scenario* (Fig. 2d–f), a shift from fossil fuels to climate-neutral energy carriers, further reduces the total fuel consumption. For passenger cars (Fig. 2d,e), gasoline is firstly replaced by ethanol, gradually shifting towards direct electrification dominance. This shift brings long-term ancillary benefits, as the efficient fuel economy of BEVs further reduces the total fuel consumption and CO_2_ emissions. Meanwhile, a CO_2_ reduction obligation accelerates the increase in alternative fuels consumption of buses as well as medium and heavy-duty trucks (Fig. 2f). In the short- and medium-term, biodiesel emerges as the least-cost fuel option for these vehicles, but by 2030 and 2040, the balance shifts towards electricity for buses and medium trucks, respectively. From 2040, hydrogen becomes the primary fuel used by heavy trucks.

### Participatory energy systems modeling – local scenarios analysis

**Fig. 3:**
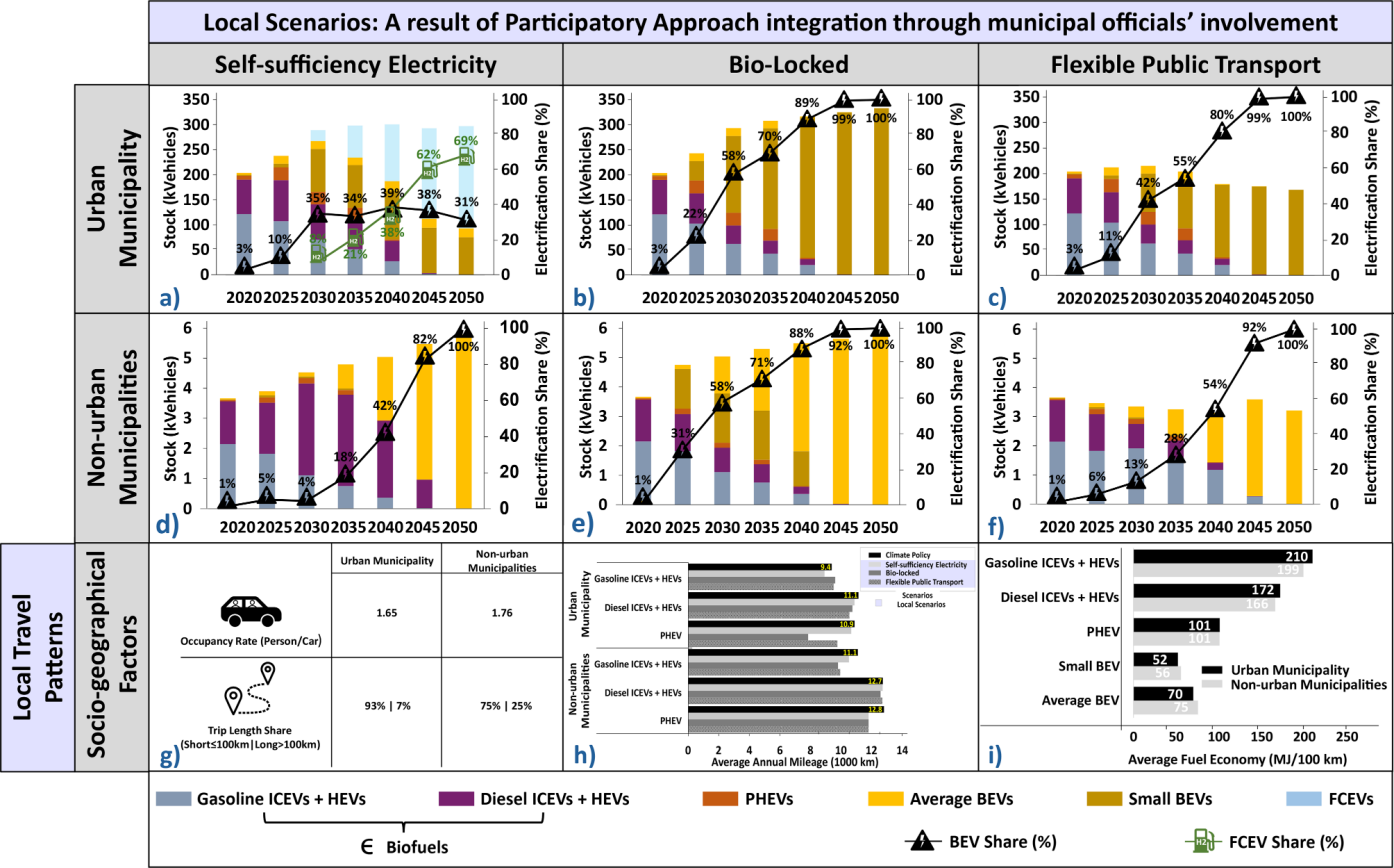
Fleet mix for different local scenarios according to the urban and non-urban municipalities (**a**-**f**). Fleet mix and electrification share in Self-sufficiency Electricity Scenario: (**a**) urban municipality; and (**d**) non-urban municipalities. Fleet mix and electrification share in Bio-Locked Scenario: (**b**) urban municipality; and (**e**) non-urban municipalities. Fleet mix and electrification share in Flexible Public Transport Scenario: (**c**) urban municipality; and (**f**) non-urban municipalities. Local travel patterns, as a result of socio-geographical context-specific factors, are presented (**g**,**h**). The occupancy rate of a passenger car and the trip length share, as modeling input data, is presented for both municipalities in Table (**g**). The average annual mileage, as a modeling result, is presented for conventional ICEVs and HEVs as well as PHEV, according to the two types of municipalities (**h**). The values dismissed small- and average-BEVs, as these vehicle types show similar values to the Climate Policy Scenario. The average fuel economy, as a modeling result, is presented according to the two considered municipalities (**i**). Biofuels – biogas and ethanol – are considered in the “Gasoline ICEVs + HEVs” category and biodiesel (HVO100) is considered in “Diesel ICEVs + HEVs”. Detailed information about model constraints, parameters, and related assumptions of the local scenarios are further described in Supplementary Information (see “Local Scenarios”). BEVs, battery electric vehicles; FCEVs, fuel cell electric vehicles; ICEVs, internal combustion engine vehicles; HEVs, hybrid electricity vehicles; PHEVs, plug-in hybrid vehicles﻿.

Given the local dimension and participatory modeling approach of this study, passenger cars are the primary focus for testing the considered local scenarios. The local scenarios added in this modeling exercise reflect different local travel patterns that are argued to differ according to different socio-geographical characteristics (see Fig. 3g–i). Furthermore, local scenarios reflect municipal officials’ ambitions on contributing towards meeting the climate goals, locally. Thorough insights into the considered local scenarios are provided in the *“*Method*”* Section and Supplementary Information appended to this study (see *“*S1.4.8. Model Scenarios*”*).

Results for the *Self-sufficiency Electricity Scenario* (Fig. 3a,d), where municipalities are assumed to supply their electricity demand with locally produced electricity from variable renewable energy sources (VRESs) – wind and solar power – show a notable shift in fleet dynamics compared to the *Climate Policy Scenario* (Fig. 1d,e). In the urban municipality (Fig. 3a), fleet electrification exceeds the local installed VRES capacity, making ICEVs running on biodiesel (HVO100) the most cost-effective option until 2030. After 2030, FCEVs are introduced in the market, becoming more cost-effective due to the existing Swedish taxes on new ICEVs registration. Post-2040, FCEVs represent over 50% of the urban fleet. In non-urban municipalities (Fig. 3d), local VRES capacity also falls short of supporting full electrification, leading to that biodiesel (HVO100) ICEVs remains optimal in the short-term. Following the 2035 ban on new ICEV sales, the model shifts to average BEVs, with investments in additional VRES capacity. Differences between the fleet composition of the two municipalities types stem from disparities in land availability for VRES expansion. Due to land restrictions, only non-urban municipalities can rely on local VRES to support BEVs, motivating the import of biofuel and hydrogen in urban municipalities. Post-2040, non-urban municipalities are characterized by a major fleet electrification, motivated by BEVs’ resulting electricity demand being met locally by VRESs, which is propelled by an average marginal electricity cost 31% lower than the corresponding cost in the *Climate Policy Scenario*.

Results for the *Bio-locked Scenario* (Fig. 3b,e) show that limiting the biofuel – biogas, ethanol, and biodiesel (HVO100) – supply, accelerates the electrification of passenger car fleets in both municipalities. The model prioritizes the allocation of limited biofuels to transport sectors, such as road freight transport, where longer driving distances benefit significantly from the high energy density of these alternative fuels. Despite significant electrification across both municipalities, the fleet composition differs from that in the *Climate Policy Scenario* (Fig. 1d,e). Post-2025, the biofuel constraint accelerates the adoption BEVs, accounting for over 50% of the total passenger car fleet in both municipalities. Small BEVs dominate the fleet of the urban municipality. In non-urban municipalities, small BEVs initially replace ICEVs running on biodiesel, as a transitional solution. However, by 2030, average BEVs achieved near cost-parity, with only a 5% cost difference compared to small BEVs, solidifying their role in the fleet composition of non-urban areas.

Results for the *Flexible Public Transport Scenario* (Fig. 3c,f) show that shifting from passenger cars to public transport is essential to achieving road transport decarbonization goals at local levels. This shift reduces the total passenger cars’ travel demand, providing flexibility to the existing fleet, and allowing it to operate longer distances and periods throughout its lifetime. Despite a lower fleet investment need, the model preferences still balance in favor of low upfront costs in urban municipalities and low total driving costs in non-urban municipalities. In both urban and non-urban municipalities, the model initially favors conventional ICEVs with a gradual change towards direct electrification. These results align with the *Climate Policy Scenario*’s findings (Fig. 1d,e), though on a smaller scale due to reduced passenger car transport demand in this local scenario.

In general, modeling local scenarios indicates that electrification appears to be the predominant decarbonization path for passenger cars. Still, the pace of electrification can be accelerated by restrictive policies on new ICEVs investments and biofuel cross-sectoral competition. Conversely, a modal shift provides additional flexibility to light-duty vehicles’ decarbonization, indirectly delaying fleet electrification.

**Fig. 4:**
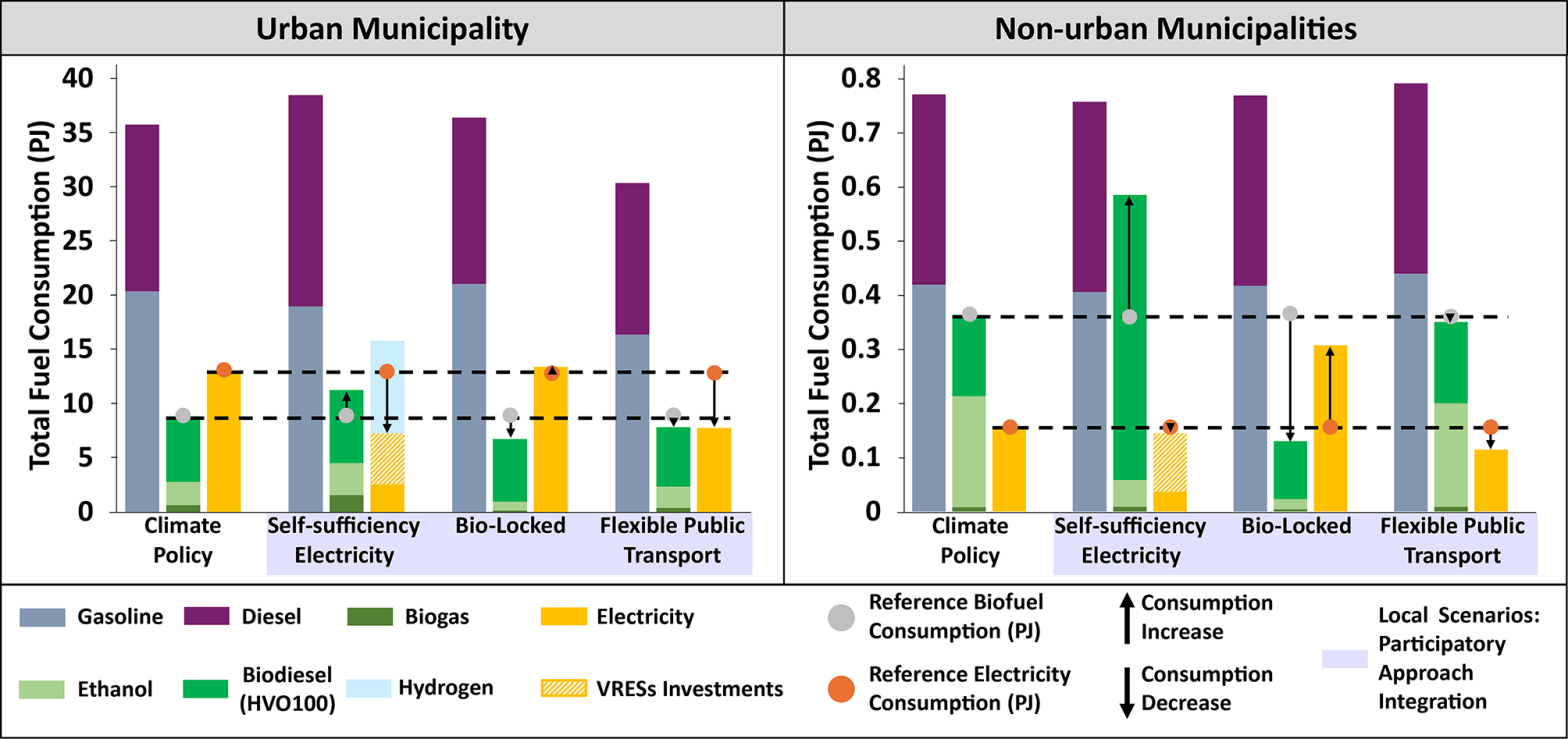
Cumulative fuel consumption (PJ) across all modeled years is presented for the climate policy scenario and each of the three local scenarios (represented in puple), for urban (**a**) and non-urban (**b**) municipalities. Detailed information about model constraints, parameters, and related assumptions of the local scenarios are further described in Supplementary Information (see “Local Scenarios”). HVO hydrotreated vegetable oil, VRES variable renewable energy source.

Quantitatively evaluating the local scenarios revealed notable differences in total fuel consumption, in both urban (Fig. 4a) and non-urban (Fig. 4b) municipalities compared to the *Climate Policy Scenario*.

In urban areas, the *Self-Sufficiency Electricity Scenario* constrains passenger car electricity demand to be met through local renewable electricity production, leading to a reduction in total electricity consumption. In the urban municipality, this shortfall is initially replaced by an increase in biofuel imports and later by hydrogen imports. In non-urban municipalities, ICEVs are identified as the most cost-effective solution until 2035, resulting in higher biofuel consumption, particularly biodiesel. After 2035, the transition to electrification accelerates, accompanied by significant investments in local VRES capacity.

The *Bio-Locked Scenario*, which imposes regional biofuel supply limits, drives higher electricity consumption in both urban and non-urban areas. This effect is more pronounced in non-urban municipalities, where biofuels were initially intended to play a transitional role in the *Climate Policy Scenario*.

By reducing the demand for passenger car transport, the *Flexible Public Transport Scenario* decreases the total fuel consumption, highlighting the potential of modal shifts in lowering energy use and thus, the resulting emissions.

## Discussion

This study primarily assesses how different socio-geographical contexts influence the composition of vehicle fleets and fuel choices, focusing on light-duty vehicles, buses, as well as medium and heavy-duty trucks. This research employs a novel approach by incorporating local spatial dynamics into a cost-optimization TIMES model, as modeling local socio-geographical scenarios, previously identified by de Oliveira Laurin et al.^53^.

This study contributes to a broader dialogue on the importance of engaging local authorities in their complex task of decarbonizing road transport. Although results are generated from municipalities in Western Sweden, the mechanisms, driving the model results, can be applied elsewhere, in both national and international socio-geographical contexts. Insights hold relevance for informing policy-making decisions worldwide on how to advance sustainable transport and mobility practices. Specifically, the findings underscore the significance of tailoring decarbonization strategies to specific local contexts rather than adopting standardized approaches. A PA, in the form of collaborating closely with local authorities and municipal officials, can facilitate a more comprehensive and inclusive discussion, which is crucial for the success of not only decarbonizing road transport but also any kind of energy transition. By adopting a local and PA perspective, this study underscores the contributing role of local authorities in advancing sustainable objectives. Specifically, local authorities provide insights into socio-geographical dynamics, enabling fair deliberation on actionable strategies. Within the scope of this study, adopting a PA enhances decarbonization decision-making, thereby contributing to (i) achieving a fossil-free Society (SDG 7); (ii) fostering a sustainable, operational, and resilient development of cities’ living areas and their services (SDG 11); and (iii) establishing inclusive and reliable transport system, as one important climate action (SDG 13). Such results align with the European Commission’s agenda^57^, recognizing the frontier role that municipalities and local communities play in driving a sustainable and more inclusive transition.

Main insights from model results on vehicle technologies and fuel mix include that road transport electrification emerges as one of the predominant strategies for decarbonization, a perspective consistent with existing literature^16–19^. Nevertheless, this study contributes to the existing body of research by delving into how the dynamics of road transport align with the logic of cost-optimization models. Such a rationale indicates that the optimal choice between upfront cost and more efficient technologies changes depending on different travel patterns. Travel patterns (e.g. annual average mileage, occupancy rate, trip length, charging patterns, and fuel economy) vary according to different socio-geographical contexts, leading to different optimal fleet compositions and fuel mixes for urban and non-urban municipalities. In urban settings, where annual average mileage and trip distances tend to be low, the study suggests that engines with lower upfront costs, such as small-size BEVs, yield cost-effective solutions. The emphasis shifts towards average-size BEVs in non-urban municipalities characterized by long trip distances. This choice not only enhances fuel economy but also reduces overall driving costs. An increasing electrification of buses as well as medium and heavy-duty trucks is also driven by their high annual average mileage patterns. Biofuel, particularly biodiesel, was identified as playing a moderate role in the short to medium-term. The flexibility of biodiesel contributes to the adaptability of the fleet dynamics during the transition period. Findings from this model exercise also suggest that municipalities could implement complementary actions alongside road transport decarbonization efforts, such as promoting a modal shift towards increased public transport adoption. This shift reduces the overall demand for passenger car transport in both municipalities, facilitating progress towards local and national CO_2_ reduction targets. Moreover, the modal shift introduces additional flexibility to the model, thereby reducing the necessity for extensive fleet replacement measures.

It is important to acknowledge that there are many uncertainties when modeling energy systems transitions, in general, and by combining a PA framework with an ESOM exercise, in particular, a message previously advocated in prior research^6,52^. To overcome some of these uncertainties, this study introduces a novel stance by implementing local socio-technical scenarios analysis. These scenarios, co-developed through local authorities and municipal officials’ participation, vividly depict the intricacies of various socio-geographical contexts, shedding light on the challenges and opportunities entailed in meeting both local and national climate targets. Overall, local scenarios revealed to accelerate the electrification of light-duty vehicles. However, this transition necessitates a resilient electric grid capable of meeting the increasing demand for electricity from a renewable perspective. In urban municipalities, it was shown that constraints on available land can challenge the electrification of light-duty vehicles at the local level. This model only considered solar parks, dismissing private roof-top solar panels as part of the local energy system. This assumption is affecting the cost-optimal fuel mix, in the urban municipality, in the *Self-sufficiency Electricity Scenario*, where biofuels and hydrogen are shown to be a cost-effective solution. In future, local energy systems may be developed including roof-top solar panels, which may lead to cost-effective fuel choices containing less biofuels and more electrified transport in urban areas. Road transport was, further, assumed as a stand-alone system, an assumption that could overvalue the biofuel role in the transport. Future work, including other transport segments (e.g., shipping and aviation), local air quality standards, dismissing national blending regulations, and European efforts toward limiting the driving of conventional ICEVs within city centers, likely would decrease the reliance of road transport on biofuels. Moreover, the modal shift to public transport assumed buses to have the same size and occupancy as the existing buses, which is a simplification that dismisses the possibility that a future public transport system may have developed. Further, travel patterns were primarily based on annual average values, potentially overlooking the nuances of daily and seasonal travel behaviors. Also, the consideration of charging profiles in this study was somewhat constrained by an approach that limited daily mileage, potentially not fully capturing evolving BEVs’ charging behaviors. Similarly, no intangible costs that might affect the investment choice of new vehicles were considered, a possible add-in to future work, as testing different hurdle rates.

Important limitations from analyses based on ESOM are connected to the fact that the model heavily depends on input data, especially regarding assumptions on the upfront costs of emerging technologies (e.g., BEVs and FCEVs), as well as corresponding fuel costs of alternative energy carriers (e.g., electricity and hydrogen). This model’s annual resolution may present challenges in accurately capturing the intermittency associated with a high penetration of renewable energy sources. This limitation could be addressed in future work by developing a model with higher temporal resolution. Additionally, this study focused solely on carbon emissions arising from the use of various energy carriers in road transport, excluding emissions from other stages of technology components’ full life cycle. This limitation could be addressed in future research by soft-linking Life Cycle Assessment (LCA) with ESOM. Model limitations, further, include that the description of the energy system is a simplification of reality in at least five important respects: (i) consideration of limited number of technologies and municipalities; (ii) assumption of price-inelastic demand; (iii) selections made only on the basis of cost; (iv) perfect foresight with no uncertainty of future costs, climate targets, or energy demand; and (v) no consideration of the importance of energy security, local air quality, or other benefits of new technologies. Consequently, the model outcomes may present “knife-edge solutions”, neglecting unexpected or immature decarbonization solutions and thus potentially lacking a comprehensive perspective. The model is, nevertheless, a useful tool to understand the system behavior and the interactions and connections between energy technology options in a future carbon-constrained world.

## Method

This section describes the developed TIMES model architecture, with a special focus on how the road transport sector was introduced into the modeling framework. The modeling framework is further described as incorporating a PA, serving as a crucial tool for defining model scenarios – local scenarios –developed collaboratively with stakeholders (i.e., municipal officials). Furthermore, and to better depict the studied local socio-geographical contexts (i.e., urban and non-urban municipalities), key model constraints, parameters, and related assumptions and scenarios are also outlined. Detailed information about the method developed, and applied, in this study, as well as input data can be found in Supplementary Information (see *“*S1.2. Modeling Framework – TIMES Model*”*, *“*S1.3. Road Transport Sector Representation*”*, and *“*S1.4. Input Data and Modeling Assumptions” Sections).

### Modeling framework – TIMES model

A cost-optimizing model was formulated according to the TIMES (The Integrated MARKAL-EFOM System) modeling framework^11^, first developed and still updated by the Energy Technology System Analysis Program (ETSAP) at the International Energy Agency (IEA)^58^. As a deterministic Linear Program (LP) model, the TIMES model integrates a long-term modeling and dynamic sectoral perspective that clarifies how an energy system can develop cost-efficiently over time (i.e. it provides a transition perspective for the whole modeling horizon), where decisions taken at one specific time period will further impact the following modeled periods. Due to its extensive and multi-geographical scope, TIMES is used to model the energy transition of different geographical contexts (from a local to a global scale)^15^.

### Road transport sector representation

**Fig. 5:**
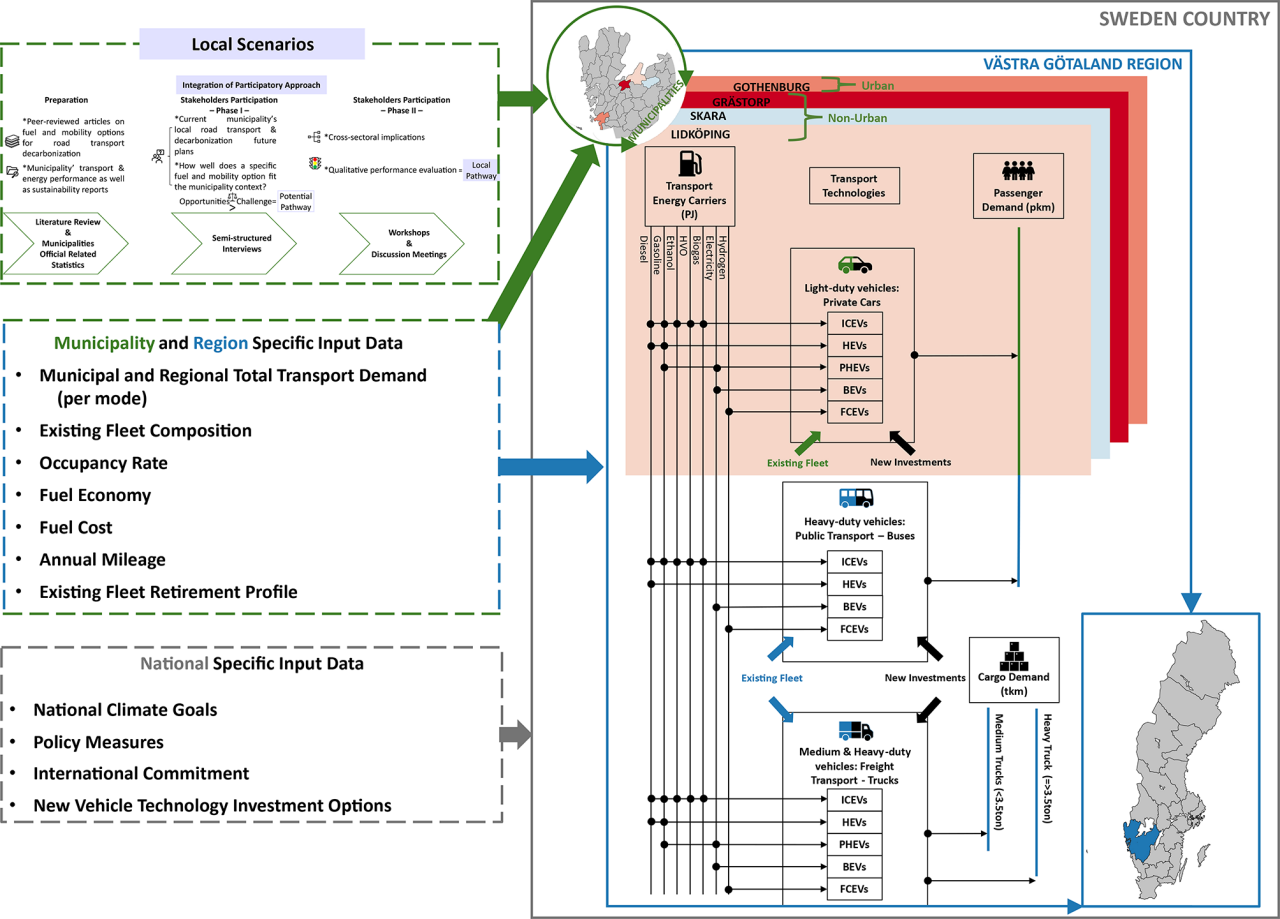
Road transport input data and model structure implemented in the TIMES model. The input data was transcribed into the model as reflecting different constraints, parameters, and related assumptions. The considered input data includes municipality-, region-, and national-specific data. These data are identified by different colors – municipality (green), region (blue), and national (grey). A distinctive soft-link was created between a PA and a cost-optimization modeling exercise by incorporating various scenarios (i.e., local scenarios) that were collaboratively developed with municipal officials. These local scenarios, derived from prior research published by the first author (see Ref.^53^), are integrated into the model to represent the specific contexts of the participating municipalities, encompassing both urban and non-urban municipalities’ specific input data (i.e., see the up left green dashed box). Municipalities and Swedish maps were generated for the specific purpose of this study, using the Quantum Geographic Information System (QGIS) 3.36.2^60^. Detailed information on the model framework – constraints, parameters, and related assumptions – as well as scenarios development and related modeling transcription are further clarified in the Supplementary Information. BEV, battery electric vehicles; FCEVs, fuel cell electric vehicles; HVO, hydrotreated vegetable oil; ICEVs, internal combustion engines; HEVs, hybrid electric vehicles; PHEVs, plug-in hybrid electric vehicles; pkm, passenger-kilometer; tkm, ton-kilometer.

The model structure for the road transport sector is described in Fig. 5. The model is run for four municipalities – one urban and three different-sized non-urban municipalities –, meaning that both existing and future investments in the private car fleet are individually modeled to meet each municipality’s total transport demand. Due to the lack of data, at the level of considered municipalities for buses and trucks, these vehicles are modeled and aggregated to better represent the whole region. This approach agrees with the typical inter-municipality demand of these two types of transport. For both private cars and buses, the total annual transport demand is expressed as activity demand passenger-kilometer (pkm), while for freight transport, demand is expressed in ton-kilometer (tkm). The road transport sector is treated as a stand-alone system and is thus the only sector modeled, following the same approach as Aryanpur et al.^18,59^. Despite being modeled as a stand-alone system, road transport is exogenously connected to the electricity sector, from which it can import electricity, at a given cost. The modeling period ranges from 2019 until 2050.

### Model scenarios

**Fig. 6:**
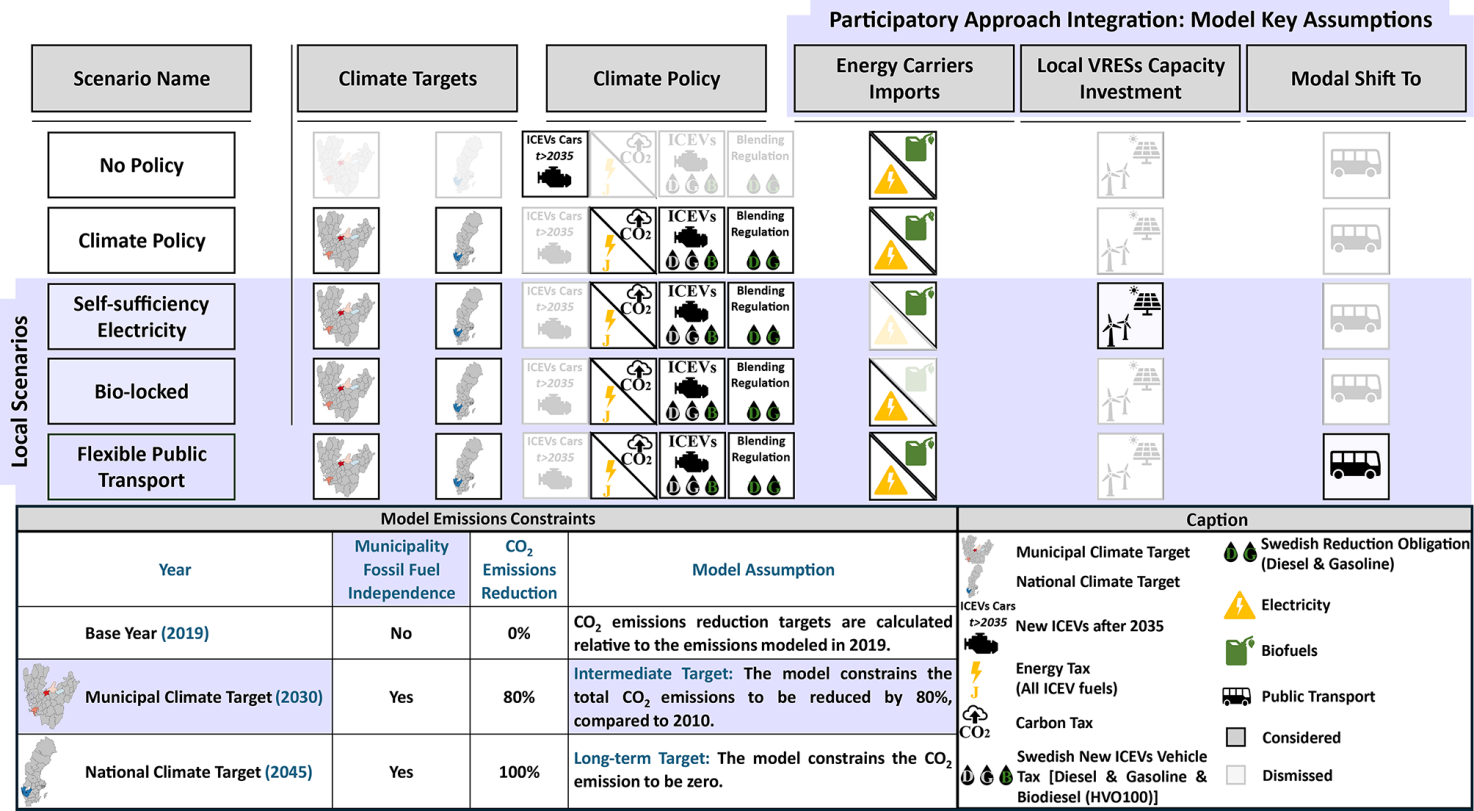
Scenario matrix and model key assumption. Local scenarios and related model constraints, parameters, as well as key assumptions, resulting from the integration of a PA as a scenario design tool in collaboration with municipal officials, are colored in purple. Under the climate policy column, the following policies are tested (i) ban on new passenger cars regarded as ICEVs after 2035; (ii) Swedish energy tax that, in combination with EU-ETS II, is added to all fossil and, from 2027, expanded to biofuel; (iii) Swedish carbon tax added to fossil fuels; (iv) increasing vehicle tax to new ICEVs passenger car registration, running on fossil fuel and biodiesel, according to Swedish “Bonus-Malus” scheme; and (v) blending regulation, according to each, every year the content of fossil fuels increased in biofuel blended share. Biofuels symbol represents biogas, ethanol (E85 for private cars and ED95 for buses as well as medium and heavy-duty trucks), and biodiesel(HVO100). Under the local VRESs capacity investment column, the electricity symbol represents renewable electricity (solar and wind). The model is constrained according to two climate targets: (i) municipal climate target, setting a reduction of 80% of emissions by 2030 compared to the 2010 level^54^; and (ii) national climate target, setting climate neutrality by 2045^55^. Municipalities and Swedish maps were generated for the specific purpose of this study, using the Quantum Geographic Information System (QGIS)) 3.36.2^60^. Detailed information on scenarios development and related constraints, parameters and assumptions are further clarified in Supplementary Information (see “S1.4.8. Model Scenarios”). ﻿ CO_2_, carbon dioxide; ICEVs, internal combustion engine vehicles; HVO, hydrotreated vegetable oil; VRES, variable renewable energy sources.

The potential impact of different socio-geographical contexts on road transport decarbonization was assessed by testing five different CO_2_ reduction scenarios, as portrayed in Fig. 6:

- *No Policy:* A baseline scenario that reflects current energy consumption trends and technological performance, without any climate change mitigation measures (i.e. policies and goals). It provides a reference point for assessing future challenges and advancements as suggested by the other scenarios.
- *Climate Policy*: Adds current and planned climate mitigation measures. At the local level of the participating municipalities, the model enforces an 80% reduction in emissions for participating municipalities by 2030, relative to 2010 levels^54^. Across all modeling levels, the net-zero emissions target must be reached by 2045^55^. To reflect current policies, the scenario incorporates Sweden’s energy and carbon taxes^61–64^, the “Bonus-Malus” vehicle tax^65^, and reduction obligations^66^ at both the national and municipal levels. As future policies, the model integrates the projected European ban in ICEVs, as forbidding this category to be part of new passenger cars sales after 2035^67^.
- *Local Scenarios*: A local perspective is added to the modeling exercise by including three different local scenarios. These scenarios were incorporated as modeling features, resulting from soft-linking a PA with an energy systems cost-optimization modeling exercise. Incorporating local scenarios, as an outcome of municipal officials’ collaboration, added a new analysis on the potential local futures of the participating municipalities. The considered local scenarios are formulated according to participatory guidelines and identified pathways presented by de Oliveira Laurin et al.^53^. The transcription of local scenarios into the modeling framework is described in the Supplementary Information. The three considered scenarios are:
- *Self-sufficiency Electricity*: It requires that the passenger cars’ resulting electricity demand, on an annual average basis, is met locally, through wind and solar power. Yet, if the local existing electricity production limit is met, the model can invest in new VRESs. This scenario provides an assessment of fuel shift, meaning that the model will choose between (i) investing in local VRE production, to meet the local passenger car electricity demand; or (ii) shifting passenger cars’ demand towards another fuel.
- *Bio-locked*: This scenario limits the availability of biofuels for biofuel road transport to what is regionally produced. Moreover, within the Västra Götaland region, only biogas and biodiesel (HVO100) are regionally produced, meaning that ethanol (E85 and ED95) is disregarded as a fuel option in this scenario. Accordingly, the model tests the regional cross-allocation of the existing biogas and biodiesel among the three road transport segments. Such an allocation is expected to highlight the monetary willingness of the different road transport segments to utilize these biofuels.
- *Flexible Public Transport*: This scenario is modeled by exogenously incorporating a modal shift from passenger cars to public transport. According to the regional agenda for public transport, this modal shift is modeled as reducing the demand for passenger car transport in the same proportion as the demand for bus passenger transport increases.

Overall, a more thorough model description, input data, and assumptions can be found in the Supplementary Information.

## Supplementary Information

Supplementary Information.

## Data Availability

The TIMES model and data used or analyzed during this study are included in this published article (and its Supplementary Information files). Further requests for materials should be addressed to M.O.L.
