# Supplementary material for: Exploring the applicability of “One-Size-Fits-All” road transport decarbonization strategies: a participatory energy systems modeling comparison of urban and non-urban municipalities
Source: Sci Rep. 2025 Mar 28;15:10747. doi: 10.1038/s41598-025-94579-w (PMC11953318; doi:10.1038/s41598-025-94579-w)
Supplement: Supplementary file 1 — Supplementary Information. [file 41598_2025_94579_MOESM1_ESM.docx]

**Exploring the Applicability of “One-Size-Fits-All” Road Transport Decarbonization Strategies: A Participatory Energy Systems Modeling Comparison of Urban and Non-Urban Municipalities**

Maria de Oliveira Laurin^1,^*, Vahid Aryanpur^2,3^, Hadi Farabi-Asl^4^, Maria Grahn^1^, Maria Taljegard^5^, Karl Vilén^6^

^1^Chalmers University of Technology, Department of Mechanics and Maritime Sciences, Gothenburg, 412 96, Sweden

^2^University College Cork, SFI MaREI Centre for Energy, Climate and Marine, Environmental Research Institute, Cork, Ireland

^3^University College Cork, School of Engineering and Architecture, Cork, Ireland

^4^RISE Research Institutes of Sweden, Division Built Environment, System Transition, Gothenburg, 412 58, Sweden

^5^Chalmers University of Technology, Department of Space, Earth, and Environment, Gothenburg, 412 96, Sweden

^6^IVL Swedish Environmental Research Institute, Gothenburg, 400 14, Sweden

^*^Corresponding author. [maria.laurin@chalmers.se](mailto:maria.laurin@chalmers.se)

**Supplementary Information**

1. **Method**

The method approach applied and developed in this study is thoroughly described in this document, defining both the studied system and its boundaries as well as introducing the developed modeling framework. Modeling key input data and scenarios’ assumptions are also presented.

- 1. **System Definition**

This study considers the development of the road transport system in the Västra Götaland region, with a special focus on the four Swedish municipalities presented in Figure S1, i.e., three non-urban municipalities – Lidköping, Skara, Grästorp –, and one urban municipality – Gothenburg.

The distinction between non-urban and urban municipalities is in line with the classification suggested by the Swedish Association of Local Authorities and Regions (SALAR)^1,2^, which describes a given municipality according to the number of municipality inhabitants and their working travel needs. Within the Västra Götaland region, four different types of municipalities are characterized by SALAR according to the following terminology: *mindre stad/tätort* (small cities); *pendlingskommun nära mindre stad/tätort* (commuting municipalities near a small city); *pendlingskommun nära större stad* (commuting municipalities near a medium-sized city); and *storstäder* (larger city level). In this study, only a “*storstäder*” municipality is considered to be urban, with the remaining types of municipalities being described as non-urban.

To better capture socio-geographical diversity, this study considers one representative of the four different municipality types: Lidköping - a small city (a municipality with a minimum population of 15,000 within the largest urban area); Skara - a commuting municipality near a small city (a municipality in which either >30% of the working population commutes to work from a small city or >30% of the employed during daytime population resides in a different municipality); Grästorp – a commuting municipality near a medium-sized city (a municipality in which >40% of the working population travels to a medium-sized city for work); and Gothenburg - a larger city level (which are municipalities with at least 200,000 inhabitants in the largest urban area).


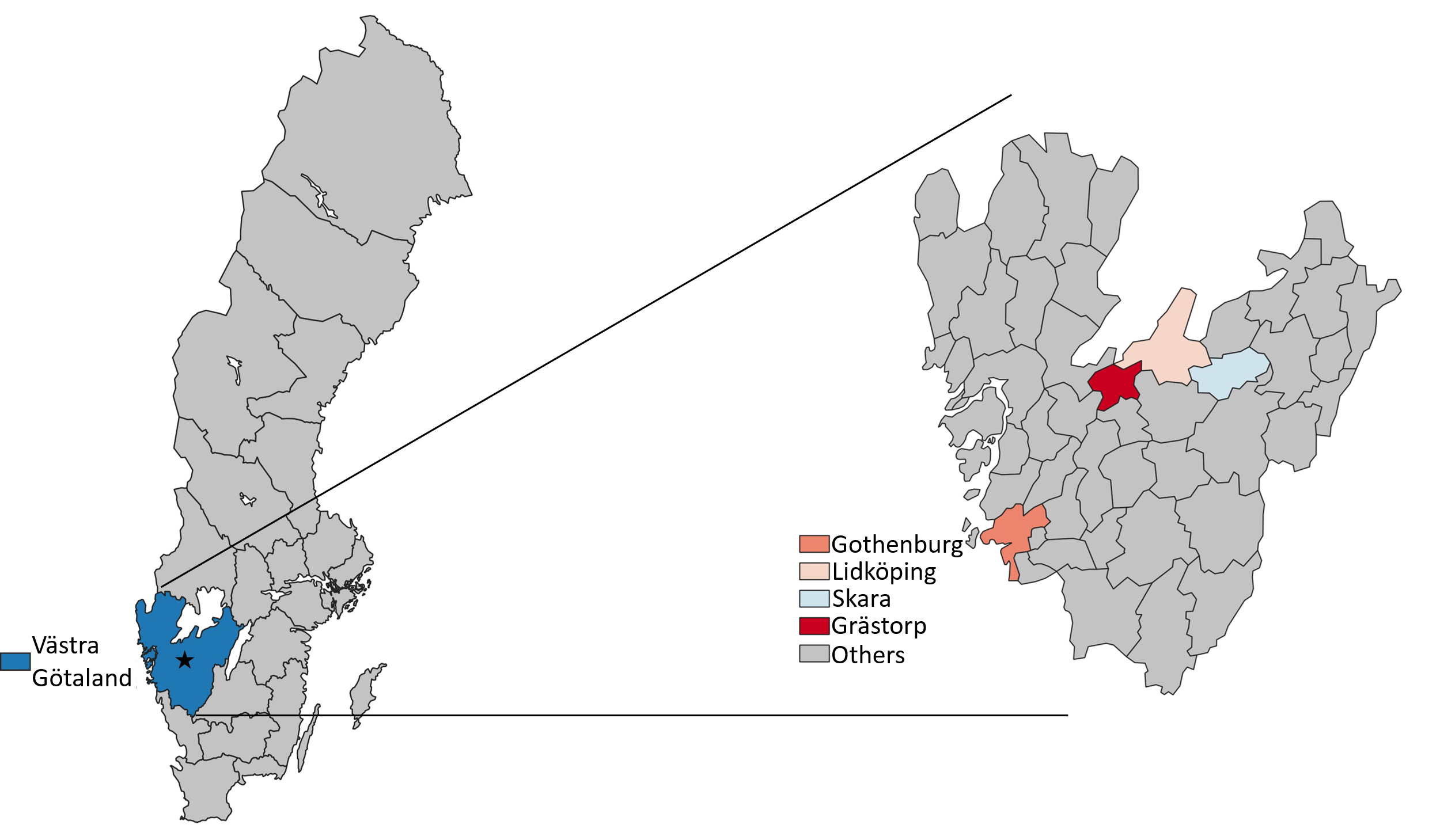


Figure S1 – On the left side, the Swedish map is presented, with the region Västra Götaland being colored blue map. On the right side, the region Västra Götaland is presented, with the municipalities covered in this study being identified with orange, red, pink, and light blue. The regions and municipalities not considered in this study are colored in grey. Municipalities and Swedish maps were generated for the specific purpose of this study, using the Quantum Geographic Information System (QGIS) 3.36.2^3^.

1. **Modeling Framework – TIMES Model**

The objective of the developed TIMES model is to find the lowest cost configuration to meet future transport demands for the whole modeled time horizon. The total cost of the system is calculated as Equation (1) follows:

|  | $NPV= \sum_{r=1}^{R} \sum_{y\in YEARS} {(1+d_{r,y})}^{REFYEAR-y}*ANNCOST (r,y)$ | (1) |
| --- | --- | --- |

The net present value, NPV, is the total cost that is minimized. ANNCOST (r,y) is the annual cost for a given region r in year y. In the specific case of the model here presented, the term ANNCOST (r,y) includes (i) annual investment costs in vehicles and infrastructure (i.e., refueling and charging stations), (ii) fixed and annual operation and maintenance (O&M), and (iii) exogenous fuel import costs (i.e., fuel cost exempt from taxes, yet including production, transportation, and distribution costs). All the considered costs are discounted, for each of the chosen periods, within the modeling horizon, in relation to a reference year. The term d_r,y_ is the discount rate, assumed to be 4%^4^, while REFYEAR is the discounting reference year (in this study considered to be 2019). The term R represents the set of regions (r) that are investigated. This study looks at differences between municipalities of the same region, the Västra Götaland region. Accordingly, in this study, five modeled regions are considered – four municipalities and the Västra Götaland region. Modeling the entire region is used when assessing public transport and freight transport (medium and heavy-duty trucks).

As described by Bunch et al.^5^, TIMES, as a bottom-up model, is, technology-explicit, allowing for a thorough representation of the energy system under study. It includes both the energy carriers and energy end-use technologies in the investigated sector(s), as well as the techno-economic interactions between them. Moreover, TIMES is defined as a partial-equilibrium model, specially developed to support the techno-economic analysis of a given energy sector, but also can include all energy sectors (see e.g.,^6–8^).

According to the above description of the TIMES model framework, technology-related data is provided to the model in terms of cost such as investment, operation and maintenance, and fuel cost, but also technical properties such as lifetimes and efficiencies of the different vehicle technologies. Two decision variables considered in the TIMES model: (i) the capacity addition, CAP (p,r,t), which represents the investments in a specific vehicle (technology) p to be made in a given region r, in a time period t; and (ii) the transport demand, GEN (p,r,t), generated by vehicle (technology) p, in region r, and time period t.

The total demand D (r,t) to be satisfied in region r and time period t, is exogenously given to the model, as a parameter. As portrayed in Equation (2), the total transport demand is set according to a load balance model constraint. Such a constraint requires that the total transport demand is met at all investigated time steps and thus this equation guarantees this balance.

|  | $\sum_{p} GEN \left( p,r,t \right)\geq D \left( r,t \right)$ | (2) |
| --- | --- | --- |

Additionally, the TIMES model uses perfect foresight, which means that the exact demands and costs for all modeled timesteps are known. This feature allows the present model to optimize between either using the existing fleet or investing in new vehicles to achieve the lowest system cost. To account for the remaining investments’ values in the end of the modeling period, a salvage value (i.e., the expected value for what a good can be sold) is also present for all transport technologies (private cars, buses, and trucks) with a remaining lifetime at the end of the modeling horizon. Such a value makes investments into these technologies at the end of the modeling period economically viable.

The framework assumed as well as input and assumptions implemented in the TIMES model specifically developed in this study will be thoroughly described in the following Sections.

1. **Road Transport Sector Representation**

To represent specific municipal light-duty – private car – as well as regional heavy-duty vehicles – public (buses) and freight (medium and heavy-duty trucks) transport – characteristics, the following parameters are set as model input data (see top left green-blue dashed outlined box in Figure S2): (i) total transport demand per mode, (ii) existing fleet composition (i.e., the existing number of each type of vehicle technology in the base year – 2019), (iii) occupancy rate (i.e., how many passengers/tons per vehicle technology), (iv) fuel economy (i.e., how many kilometers is possible to drive per each unit of fuel consumed, measured in Mkm/PJ), (v) fuel cost (i.e., cost of the whole fuel value chain composed by fuel production, fuel distribution, and fuel infrastructure), annual mileage (i.e., the average driven distance by a vehicle technology, measured in km), and (vi) existing fleet retirement profile (i.e., the probability of a vehicle technology reaching the end of its lifetime). In the case of light-duty private cars, these parameters represent mean values for each municipality, whereas for both public transport and freight, represent mean values for the Västra Götaland region. Such input data is thoroughly presented in Sections S1.4.

The number of each vehicle technologies are implemented in the model as a parameter (i.e., existing stock), but also as a decision variable (i.e., future investment choices). A given vehicle technology, according to its engine type, consumes (in PJ) a specific transport fuel. Five different vehicle technologies are considered: internal combustion engine vehicles (ICEVs), hybrid electric vehicles (HEVs), plug-in hybrid electric vehicles (PHEVs), battery electric vehicles (BEVs), and fuel cell electric vehicles (FCEVs). Similarly, seven different energy carriers (in this study, for simplicity reasons, called fuels) are considered in the model: diesel, gasoline, biogas, ethanol (E85/ED95), hydrotreated vegetable oil (HVO100), electricity, and hydrogen. The choice of which vehicle technologies and fuels options to consider in the model was based on both today’s existing fleet (2019), as well as technologies that do not exist in today’s road transport fleet but have the potential to enter the market within the model’s time horizon (i.e., from 2019 to 2050). Hydrogen is considered to be an energy carrier option in the model from the year 2030, while all other fuels can be used from 2019.

Municipal and regional commitments, aiming for a reduction of greenhouse gases (GHG) emissions as an action to mitigate climate change (in this study determined as “climate commitment”), are added to the model as constraints (e.g., the considered municipalities aim to become “fossil fuel independent” by 2030^9^ - read more in Section SS1.4.6). Different national CO_2_ reduction strategies and international commitments are further implemented in the model (see bottom left grey dashed box in Figure S2).


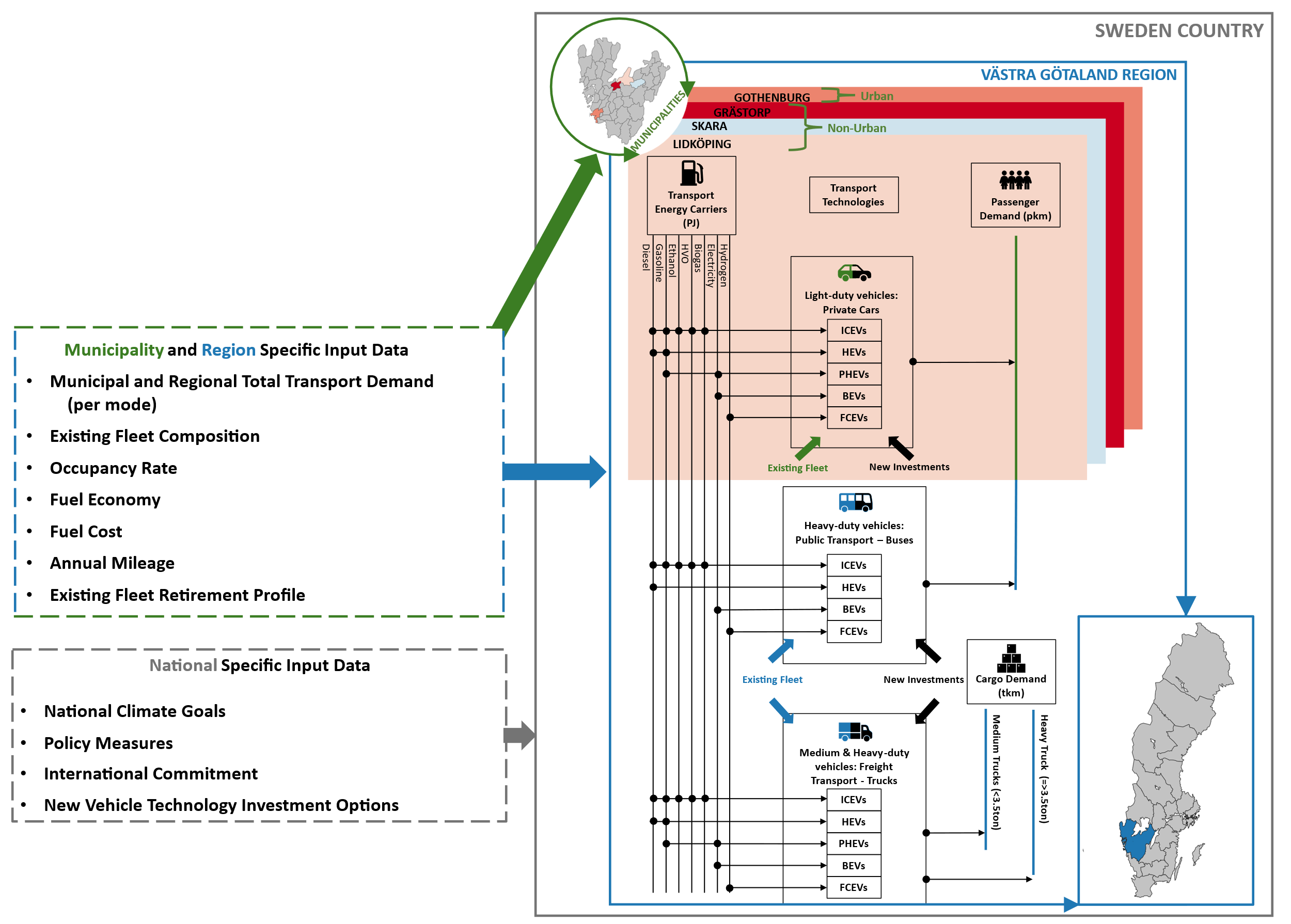
Figure S2 – Road transport input data structure implemented in the TIMES model. The model includes three types of road transport vehicles: light-duty vehicles – private cars – as well as medium and heavy-duty vehicles – public transport (buses) and freight transport (trucks). Private cars are modeled at the local level of four different municipalities; both public transport and freight transport are modeled at the regional level of the Västra Götaland region. The data implemented in the model includes municipality-, region-, and national-specific data. These data are identified by different colors – municipality (green), region (blue), and national (grey). Municipalities and Swedish maps were generated for the specific purpose of this study, using the Quantum Geographic Information System (QGIS) 3.36.2^3^. HVO, hydrotreated vegetable oil; ICEV, internal combustion engines; HEV, hybrid electric vehicles; PHEV, plug-in hybrid electric vehicles; BEV, battery electric vehicles; FCEV, fuel cell electric vehicles; pkm, passenger-kilometer; tkm, ton-kilometer.

1. **Input Data and Modeling Assumptions**

A TIMES model is based on a set of input data and modeling assumptions about the main drivers of the energy system under study. Both input data and assumptions are often included in the model exogenously. The key input data and modeling assumptions considered in this study will be presented in the following subsections.

1. **Timeframe**

The presented TIMES model was firstly developed according to the timeframe of meeting municipalities' climate commitment, “*Klimat 2030*” agreement (i.e., regional Swedish climate target signed by Västra Götaland municipalities to meet fossil-fuel independence, defined by them as reducing their GHG emissions by 80%, comparatively to 1990, at a domestic level), by 2030^9^. The modeling period, however, was extended until 2050. Accordingly, and due to its dynamic perspective, the model can capture how the system evaluates from meeting the local fossil-fuel independence in 2030 to achieving the national climate goals in 2045^10^. To better capture the transition path, the period from the base year 2019 up to 2030 is modeled in one-year time steps. From 2030, the main focus of the model was to monitorize the system (i.e., to understand how the studied system, after meeting a given goal, will look in the long-term) and thus, the model applies five-year time steps.

1. **Transport Demand**

**Base Year**

No official statistics on the future passenger and freight transport demands of the four municipalities in this study were found nor for the considered region. Accordingly, the estimation of future transport demands in this study has been made by merging different sources and data. For private cars and buses, as expressed in Equation (3), the base year (2019) demand was calculated by multiplying the total number of vehicles of each municipality (cars) and region (buses) by the occupancy rate and the vehicle’s annual mileage:

| $Passenger Transport Demand \left( pkm \right)=$  $Total number of registered vehicles*[Occupancy rate \left( number of people \right)*Annual Mileage \left( km \right)](per vehicle)$ | (3) |
| --- | --- |
|  |  |

The total number of cars in each municipality and the number of buses in the Västra Götaland region, registered in 2019, was directly derived from the Swedish Vehicle Statistics Database^12^. The car occupancy was extrapolated from the values suggested by the Swedish Transport Administration (Trafikverket)^13^. According to Trafikverket, car occupancy varies with the trip length – for trips shorter or equal to 100 km, the car occupancy averages at 1.61 passengers/car, and for trips longer than 100 km, the car occupancy is measured to be 2.22 passengers/car. Within the same country, car occupancy has been measured as differing between different socio-geographical contexts (see, e.g.,^14^). Given the scope of this study, the car occupancy resolution was expanded to better capture how these values vary according to the four different modeled municipalities. Accordingly, from the open synthetic population data on Swedish activity-travel patterns^15^, it was possible to calculate, for the different municipalities, the share of short trips (shorter or equal to 100 km) and long trips (longer than 100 km). Calculating municipalities’ short and long trip shares allowed for calculating the weighted average car occupancy per municipality, as expressed in Equation (4):

| $Occupancy rate \left( number of people per vehicle \right)=share of short trip \left( \% \right)*1.61+share of long trip \left( \% \right)*2.22$ | (4) |
| --- | --- |

According to Equation (4), the car occupancy was proven to differ between the different types of municipalities defined by SALAR – Lidköping: 1.70; Skara: 1.70; Grästorp: 1.82; Gothenburg: 1.65.

For buses, the occupancy rate was assumed to be 9.13, as implemented by the ON-TIMES energy system model open source^16^. The average annual mileage for both private cars and buses follows the Swedish national travel survey^17^ and Länsstyrelserna^18^. The different values used to calculate the passenger transport demands are described in Table S1 and Table S3.The average annual mileage is also applied to the new investments of both private cars and buses. In the particular case of the existing stock of private cars, different engines are defined with different annual mileage, according to the national survey^17^ (ref), as described in Table S2. For both new invested small BEVs and average BEVs, the standard public charging (i.e., 7 kW) events were considered, by a 15% lower annual mileage, compared to the other passenger car types, based on the assumptions previously presented by Andersen et al.^19^.

Table S1 – Input data used to calculate private car passenger transport demand for the base year 2019. The municipalities' label colors match the scheme used in the map presented in Figure S1. The references of value directly extrapolated from open data sources are also given.

| **Municipality** | **Socio-geographical context** | **SALAR’ definition** | **Total number of registered cars** | **Trips length**^15^ | | **Car occupancy (passenger/cars)** | **Average Annual mileage (km/car)** |
| --- | --- | --- | --- | --- | --- | --- | --- |
|  |  |  |  | $\boldsymbol{\leq}$ **100 km (%)** | $\boldsymbol{>}$ **100 km (%)** |  |  |
| Lidköping | Non-urban | Small city | 22,680 | 85 | 15 | 1.70 | 11,644 |
| Skara | Non-urban | Commuting municipality near small city | 10,212 | 85 | 15 | 1.70 | 11,938 |
| Grästorp | Non-urban | Commuting municipality near a medium-sized city | 3,507 | 65 | 35 | 1.82 | 13,106 |
| Göteborg | Urban | Larger city | 189,391 | 93 | 7 | 1.65 | 11,209 |
| **References** | | ^2^ | ^12^ | ^15^ | | Equation (4) | ^18^ |

Table S2 – Annual mileage per car of the existing stock, based on the Swedish national travel survey, varying within different municipalities and cars type.

| **Passenger Cars Type** | **Existing Stock Annual Mileage (km/car)** | | | | **References** |
| --- | --- | --- | --- | --- | --- |
|  | **Lidköping** | **Skara** | **Grästorp** | **Gothenburg** |  |
| Gasoline ICEVs | 9,256 | 9,489 | 10,418 | 8,968 | ^17^ |
| Diesel ICEVs | 16,134 | 16,541 | 18,159 | 15,545 |  |
| HVO100 ICEVs | 16,134 | 16,541 | 18,159 | 15,545 |  |
| Ethanol ICEVs | 12,001 | 12,304 | 13,508 | 11,563 |  |
| Biogas ICEVs | 14,281 | 14,642 | 16,074 | 13,760 |  |
| Small BEVs | 10,850 | 11,124 | 12,212 | 10,454 |  |
| Average BEVs | 10,850 | 11,124 | 12,212 | 10,454 |  |
| Gasoline HEVs | 12,862 | 13,187 | 14,477 | 12,392 |  |
| Diesel HEVs | 12,862 | 13,187 | 14,477 | 12,392 |  |
| PHEVs | 15,282 | 15,668 | 16,074 | 14,724 |  |

For the freight transport, as displayed in Equation (5), the transport demand for the base year of both medium-duty and heavy-duty trucks was calculated following the same logic as passenger transport demand, yet expressing occupancy rate in tons of goods per vehicle:

| $Freight Transport Demand \left( tkm \right)=$  $Total number of registered vehicles* \left[ Occupancy rate \left( tons \right)*Annual Mileage \left( km \right) \right]\left( per vehicle \right)$ | (5) |
| --- | --- |

The total number of registered trucks for the Västra Götaland region in 2019 was retrieved from the Swedish Vehicle Statistics Database^12^. The occupancy rate and annual mileage were estimated to be 0.40 tons/truck for medium-duty trucks and 7.79 tons/trucks for heavy-duty trucks, aligning with the values used by the ON-TIMES energy system model open source^16^. The different values used to calculate the freight transport demand are presented in 3.

Table S3 – Input data used to calculate the bus and freight transport demand for the base year 2019. The municipalities' label colors match the scheme used in the map presented in Figure S1. The references of value directly extrapolated from open data sources are also given. NA, Non-applicable.

| **Region** | **Vehicle type** | **Total number of registered vehicles** | **Occupancy rate** | **Daily vehicle-traveled distance (km/person)** | **Annual mileage (km/vehicle)** |
| --- | --- | --- | --- | --- | --- |
| Västra Götaland | Bus | 2,683 | 9.13 (passenger/bus) | 10 | 33,325 |
|  | Medium Truck | 85,823 | 0.4 (tons/truck) | NA | 13,000 |
|  | Heavy Truck | 13,884 | 7.79 (tons/truck) | NA | 70,250 |
| **References** | | ^12^ | ^16^ | ^17^ | ^16^ |

To better capture the technical limitations that BEVs and FCEVs present when implemented as decarbonization technology solutions to buses and trucks, this study considered that the annual mileage and cargo capacity of these vehicles are lower compared to ICEVs^20,21^. For buses, the annual mileage is considered to be 28,326 km/bus-year, yet the passenger capacity remains. For medium-duty trucks, the annual mileage and the cargo capacity are defined as 10,700 km/truck-year and 0.38 tons/truck, respectively. For heavy-duty trucks, the annual mileage and the cargo capacity are defined as 55,610 km/truck-year and 6,08 ton/truck, respectively.

**Future**

The future passenger transport demands for both private cars and buses were assumed to increase annually according to the anticipated growth of the Gross Domestic Product per capita, as suggested by the prognosis presented by Trafikverket^22^. For private cars, the transport demand is expected to increase by 1.1% annually from the base year, 2019, until 2040. Post 2040, private car transport demand experiences an annual increase of 0.6% in transport demand. The bus transport demand is projected to increase by 0.6% every year over the entire modeled time. For freight transport, as assumed by Forsberg et al.^23^, an annual increase of 2.3% in the transport demand is expected. Detailed values for future passenger and freight transport demands are presented in Table S4.

Table S4 - Transport demand input data for private cars, buses, and trucks. For both private cars and buses the transport demand is given in billion (B = 10^9) pkm and for freight transport in Btkm. The input data is calibrated for the year 2022. The municipalities' label colors match the scheme used in the map presented in Figure S1.

| **Transport Demand** | | **Type** | **Units** | **2019** | **2025** | **2030** | **2035** | **2040** | **2045** | **2050** | **References** |
| --- | --- | --- | --- | --- | --- | --- | --- | --- | --- | --- | --- |
| **Municipalities** | Lidköping | Private Car | Bpkm | 0.446 | 0.452 | 0.477 | 0.504 | 0.533 | 0.549 | 0.566 | ^22^ |
|  | Skara |  |  | 0.203 | 0.209 | 0.221 | 0.234 | 0.247 | 0.254 | 0.262 |  |
|  | Grästorp |  |  | 0.806 | 0.086 | 0.090 | 0.096 | 0.101 | 0.104 | 0.107 |  |
|  | Göteborg |  |  | 2.975 | 3.648 | 3.853 | 4.070 | 4.299 | 4.429 | 4.564 |  |
| **Region** | Västra Götaland | Bus |  | 0.816 | 0.846 | 0.872 | 0.898 | 0.926 | 0.954 | 0.983 |  |
|  |  | Medium Truck | Btkm | 0.466 | 0.512 | 0.573 | 0.642 | 0.719 | 0.806 | 0.903 | ^23^ |
|  |  | Heavy Truck |  | 7.596 | 8.707 | 9.755 | 10.930 | 12.246 | 13.720 | 15.373 |  |

1. **Fuel Cost**

In this TIMES model, different fuels, as energy carriers, were assumed to be an exogenous (imported) commodity that was consumed to satisfy a specific transport demand. As an imported commodity, the available fuels are modeled with an associated cost, expressed in M€/PJ. As described in Equation (6), the fuel cost sums the different cost-components associated with three stages of the entire energy carrier supply chain – production, transmission and distribution (i.e., cost associated with the transport of the different fuels to the refueling stations), as well as infrastructure (i.e., cost associated with future investments in fuel infrastructure, such as fast public charging stations):

| $Fuel Cost \left( \frac{M€}{PJ} \right)={Fuel Cost}_{Production}\left( \frac{M€}{PJ} \right)+ {Fuel Cost}_{Transmission \& Distribution}\left( \frac{M€}{PJ} \right)+ {Fuel Cost}_{Infrastructure}\left( \frac{M€}{PJ} \right)$ | (6) |
| --- | --- |

The production cost follows the same projections as presented in Forsberg et al.^23^. Yet, the authors did not specify the cost associated with the production of biogas and biodiesel. For these two fuels, the production cost was primarily based on Energy Analyses values^24^ and later calibrated to the costs used for the other fuels (i.e., to ensure consistency in the assessment of production costs from two distinct databases, the production costs of a specific energy carrier across both databases were compared. Subsequently, these comparisons were used to readjust the production costs of biogas and biodiesel to align with the values recommended by Forsberg et al.^23^). Transmission and distribution costs included in this model were the same as the costs suggested by Energy Analyses values^24^. Both production costs as well as transmission and distribution costs did not vary between transport modes and were thus assumed to be the same for private cars, buses, and trucks.

The availability of recharging and refueling infrastructure can be a critical factor when assessing the cost of biogas, electricity, and hydrogen. The uptake of these energy carriers requires further investments in infrastructure, a factor that was taken into consideration in this model. Accordingly, this model included infrastructure costs associated with (i) biogas (i.e., future investments in refueling stations supplied by local biogas grid), (ii) electricity (i.e., future investments in public standard and fast charging infrastructure), and (iii) hydrogen (i.e., future investments in hydrogen refueling stations supplied by a tanker truck). Similarly to the production cost, the infrastructure costs combined the values suggested by both^23,24^ and were modeled as varying between different transport modes.

The three cost components are presented in Table S5.

Table S5 – Fuel cost input data used for private cars, buses, and trucks. For private cars, the electricity infrastructure cost is assumed to vary between the type of charging – standard public charging and fast public charging. Yet, buses and trucks, as transport-service modes tend to optimize their charging profiles. Accordingly, only fast charging is assumed for these transport modes and thus, the electricity infrastructure fuel cost associated with these two transport modes only captures this charging type. HVO, hydrotreated vegetable oil; E85, 85% ethanol blended in 15% gasoline; ED95, 95% ethanol blended in 5% ignition improvers; NA, non-applicable; SC, standard public charging; FC, fast public charging.

| **Fuel Type** | **Fuel Cost (M€/PJ)** | | | | |
| --- | --- | --- | --- | --- | --- |
|  | **Production** | **Transmission and Distribution** | **Infrastructure** | | |
|  |  |  | **Private Cars** | | **Buses & Trucks** |
| Diesel | 13.8 | 0.2 | NA | | |
| Gasoline | 13.8 |  |  |  |  |
| E85/ED95 | 24 |  |  |  |  |
| HVO100 | 24 |  |  |  |  |
| Biogas | 24 | 0.3 | 0.7 | | 0.7 |
| Electricity | 8.2 | 3.7 | SC | 11 | 9.5 |
|  |  |  | FC | 42.6 |  |
| Hydrogen | 18.1 | 4.7 | 22.1 | | 19.0 |
| **References** | ^23,24^ | | | | |

BEVs’ charging profiles were included by applying two distinct electricity costs (ECost): standard public charging (SP) and fast charging (FC). These two costs are presented in Table S5. The resulting electricity cost is determined using Equation (7) and Equation (8), which account for the average distribution of charging events across work (CW), home (CH), and additional unforeseen fast-charging requirements (CFC). The shares for these calculations were sourced from Andersen et al.^19^and indicate that BEVs typically charge: (i) 75% at work; (ii) 90% combined at work and home for vehicles utilizing home charging; and (iii) 10% through unplanned fast-charging events.

| *Small BEVs ECost = Share of (CW) (%) * SP ECost + (Share of CFC + Long Trips) (%) * FC ECost* | (7) |
| --- | --- |
| *Average BEVs ECost = Share of (CW + CH) (%) * SP ECost + Share CFC (%) * FC ECost* | (8) |

**S1.4.4 Emission Factors**

The different fuels, as energy carriers, are associated with different GHG emission factors, which can be included in TIMES. Yet, in this study, due to its scope, only carbon dioxide (CO_2_) emissions were considered. The emission factors were thus measured in ktonCO_2_/PJ, meaning that such emissions were embedded in the use of each fuel option. The CO_2_ emissions result from the combustion of the different energy carriers were considered. For biogenic fuel options, CO_2_ emissions have been simplified to disregard upstream emissions. For electricity, and consequently, for hydrogen, the emissions factor was based on actual values from the Swedish electricity mix between 2019 and 2022^25^. Additionally, in line with Sweden's national climate policies, the emissions factor was projected to reflect the country's 2030 reduction targets and the goal of achieving 100% renewable energy penetration by 2040^26^. Thus, the emissions related to (i) the feedstock type used in the production of fuels, (ii) the stages of extracting, converting, and distribution of the fuel chain, and (iii) the vehicle manufacturing and infrastructure – road and fuel supply infrastructure – construction, are not included in this study. Result of this assumption, pure biofuels (ethanol, HVO100, and biogas), which are considered to be biogenic, electricity (carbon-neutral electricity mix post-2040), and hydrogen (i.e., produced via electrolysis, resulting from renewable electricity post-2040) are assumed to be carbon-neutral. The different emissions factors and corresponding references are presented in Table S6.

Table S6 – Carbon dioxide emissions factors for the different fuels, as energy carriers, included in the TIMES model. In the case of fossil fuels – diesel and gasoline –, the fuel composition, according to existing national reduction quota mandates^11^ is expected to change over the modeled years. These changes are moving towards accommodating higher shares of biofuel, meaning that the emissions factors of these fuels also change over the years, where we have simplified it into two time periods: 2019-2029 and 2030-2050. FAME, Fatty Acid Methyl Esters; HVO, Hydrotreated Vegetable Oil; DS, Diesel; BDS, Biodiesel; GS, gasoline; ETH, pure ethanol; E85, 85% ethanol blended in 15% gasoline; ED95, 95% ethanol blended in 5% fossil-fuel-based ignition improvers; NG, Natural Gas; BG, Biogas.

| **Energy Carrier (Fuels)** | **Energy Carrier Composition** | **Emission Factor (ktonCO_2_/PJ) ^a)^** | | **References** |
| --- | --- | --- | --- | --- |
|  |  | **2019 - 2029** | **2030 - 2050** |  |
| Diesel | Blending share between diesel and biodiesel (FAME & HVO) | *79% DS + 21% BDS* | *34% DS + 66% HVO* | ^11,27^ |
|  |  | 58.54 | 25.19 |  |
| Gasoline | Blending share between gasoline and pure ethanol | *95.8% GS + 4.2% ETH* | *72% GS + 28% ETH* |  |
|  |  | 70.221 | 57.776 |  |
| Ethanol (E85) | Blending share between gasoline and pure ethanol used in light-duty private cars | *15% GS + 85% ETH* | | ^27,28^ |
|  |  | 11.00 | |  |
| Ethanol  (ED95) | Blending share between fossil fuel-based ignition improvers and pure ethanol used in buses as well as medium and heavy freight transport | *5% Ignition Improvers + 95% ETH* | | ^27,29^ |
|  |  | 3.50 | |  |
| Biodiesel (HVO100) | Pure hydrotreated vegetable oil | 0 | | ^23^ |
| Biogas | Blending share between natural gas and biogas | *3% NG + 97% BG* | *100% BG* | ^27,30^ |
|  |  | 2.28 | 0 |  |
| Electricity | Electricity mix | 8 (2019) / 7.5 (2020) / 8 (2021) / 7.8 (2022) / 5.3 (2030) / lowering linearly to 0 (post-2040) | | ^25,26^ |
| Hydrogen | Hydrogen | Follow the same trend as electricity | |  |

1. It should be noted that the CO_2_ emission factors are not static between 2019 and 2029 but decrease linearly from the values assumed in 2019 to the values assumed in 2030. The same rationale is applied to the values between 2030 and 2050.
2. **Transport Technology Database**

Different vehicles are set as technology options available in this model. Such a technology portfolio included both the existing vehicle stock and future investment vehicle options, as indicated in Table S7.

Table S7 - Technology portfolio included in the TIMES model, for both current existing stock and future investment options. The availability of each vehicle technology was specified by the year according to which they are available as model options. Some vehicle technologies were not part of the existing stock, yet they were assumed to be an investment option already for the base year (2019) – these options are shaded in green. Options that are part of the existing stock, yet not considered as future investment options are shaded in grey. M, medium-duty; H, heavy-duty; ICEVs, internal combustion engine vehicles; BEVs, battery electric vehicles; HEVs, hybrid electric vehicles; PHEVs, plug-in hybrid electric vehicles; FCEVs, fuel cell electric vehicles; HVO, hydrotreated vegetable oil; NA, non-applicable.

| **Road Transport Segment** | | **ICEVs** | | | | | **BEVs** | | **HEVs** | | **PHEVs** | **FCEVs** |
| --- | --- | --- | --- | --- | --- | --- | --- | --- | --- | --- | --- | --- |
|  |  | **Gasoline** | **Diesel** | **HVO100** | **Ethanol** | **Biogas** | **Small** | **Average** | **Diesel** | **Gasoline** |  |  |
| Passenger Cars | | 2019 | 2019 | 2019 | 2019 | 2019 | 2019 | 2019 | 2019 | 2019 | 2019 | 2030 |
| Buses | | 2019 | 2019 | 2019 | 2019 | 2019 | NA | 2019 | 2019 | NA | NA | 2030 |
| Trucks | M | 2019 | 2019 | 2019 | 2019 | 2019 | NA | 2019 | 2019 | 2019 | 2019 | 2030 |
|  | H | 2019 | 2019 | 2019 | 2019 | 2019 | NA | 2019 | 2019 | NA | 2019 | 2030 |

This study did not consider future driving behavior changes, meaning that travel patterns will remain the same within the modeled time horizon and thus model parameters such as (i) occupancy rate, (ii) fuel economy, (iii) annual mileage, and (iv) operation and maintenance cost, remain the same between existing stock and future invested vehicle options. It is also important to note that the focus of this study was to assess the comparative attractiveness of different engines and fuel options, within a specific road transport segment, meeting different CO_2_ emission goals. Consequently, it was not part of the scope to predict the likelihood of how fast changes in technology or fuel adoption may occur, and thus, no constraints on potential future market shares or technology deployment rates were part of the model framework.

**Private Cars**

The existing stock is described according to 10 different types of private cars. The number of registered gasoline ICEVs, ethanol ICEVs, biogas ICEVs, and PHEVs for each of the four considered municipalities is directly retrieved from Trafikanalys 2019 statistics^12^. The same data source also provides the total number of registered diesel ICEVs, BEVs, and HEVs, yet at an aggregate level (i.e., the registered diesel ICEVs include both ICEVs running on diesel and HVO100, the registered BEVs sum together both small and average BEVs, while registered HEVs are a result of summing gasoline and diesel HEVs)^12^. To split the total registered diesel ICEVs into diesel ICEVs and HVO100 ICEVs, this study applied IEA Bioenergy’s ratio^31^, suggesting that HVO100 represents 30% of the whole diesel ICEVs fleet. This study also categorized BEVs into small-size BEVs and average-size BEVs – small-size BEVs house a 20-kWh battery^4^ and average-size BEVs house a 40-kWh battery^32^ –, assuming that small-size BEVs represent 31% of the whole BEVs fleet, as previously suggested by Hagos et al.^4^. Small-size BEVs will have different needs for fast charging when compared to average-size BEVs, which in this study is implemented through different electricity costs. As presented in Table S5, different electricity costs are used to mimic the small BEVs’ need for public high-cost fast charging instead of low-cost slow charging, assumed to be sufficient for the average BEVs. No concrete data was found regarding the existing stock ratio between gasoline HEVs and diesel HEVs; thus, this study considered an equal share between diesel and gasoline HEVs for the base year. The existing stock is thoroughly presented in Table S8.

Table S8 - Existing stock for passenger cars for the base year 2019, for each of the considered municipalities. The municipalities' label colors match the scheme used in the map presented in Figure S1. The references of value directly extrapolated from open data sources are given in brackets. ICEVs, internal combustion engine vehicles; BEVs, battery electric vehicles; HEVs, hybrid electric vehicles; PHEVs, plug-in hybrid electric vehicles.

| **Passenger Cars Types** | **Existing Stock (Number of Vehicles) - 2019** | | | | **References** |
| --- | --- | --- | --- | --- | --- |
|  | **Lidköping** | **Skara** | **Grästorp** | **Gothenburg** |  |
| Gasoline ICEVs | 12,572 | 6,025 | 1,868 | 105,557 | ^12^ |
| Diesel ICEVs | 5,654 | 2,397 | 960 | 44,958 | ^12,31^ |
| HVO100 ICEVs | 2,423 | 1,027 | 411 | 19,268 |  |
| Ethanol ICEVs | 1,066 | 468 | 175 | 7,933 | ^12^ |
| Biogas ICEVs | 260 | 81 | 53 | 2,875 |  |
| Small BEVs | 20 | 5 | 2 | 332 | ^4,12,32^ |
| Average BEVs | 44 | 11 | 6 | 738 |  |
| Gasoline HEVs | 189 | 86 | 14 | 2,726 | ^12^ |
| Diesel HEVs | 189 | 86 | 14 | 2,726 |  |
| PHEVs | 198 | 28 | 5 | 2,387 |  |

As future investment passenger car options, the same ten types of vehicles were considered, with an additional option for FCEVs running on hydrogen from 2030^23^.

Vehicle occupancy and annual mileage remained constant for the different engines within the same municipality. Yet, both values were considered to change according to the different types of municipalities. These parameters, which combine different data sources^13,15,17^ are presented in detail in Table S1. Driving patterns (e.g., trip length, traffic, and distance to refueling stations) are proven to change according to different socio-geographical settings, which can be translated into fuel economy changes^33^. Accordingly, this study assumed that the fuel economy varies between different engines but also between different urban and non-urban municipalities. The fuel economy for the different engines was retrieved from the JRC-EU-TIMES database, which describes the fuel economy according to the trip length (i.e., short for distances below 100 km and long for distances of at least 100 km)^34^. JRC-EU-TIMES database does not specify the fuel economy for HVO100, ethanol, and biogas. Accordingly, this study calculated the fuel economy for these biofuels by assuming that (i) HVO100 has the same fuel economy as diesel^35^, (ii) ethanol (E85) fuel economy is 1% lower than gasoline fuel economy^35^, and (iii) biogas has the same fuel economy as compressed natural gas^36^. The fuel economy differs when a vehicle is driving short and long distances^34^. To distinguish how the fuel economy of a given engine differs between the municipalities, the weighted fuel economy was calculated according to the share of short and long trips of the different municipalities, as portrayed in Equation (9).

| ${Fuel Economy \left( \frac{Mkm}{PJ} \right)}_{Engine Y}^{Municipality X}=$  $\sum_{Engine Y}^{Municipality X} Short Trips \left( \% \right)*Short Distances Fuel Economy+Long Trips \left( \% \right)*$  $Long Distances Fuel Economy$ | (9) |
| --- | --- |

The fuel economy input data, as a direct outcome of Equation (9) is presented in Table S9.

Table S9 - Fuel economy for passenger cars for the whole model horizon. The municipalities' label colors match the scheme used in the map presented in Figure S1. ICEVs, internal combustion engine vehicles; BEVs, battery electric vehicles; HEVs, hybrid electric vehicles; PHEVs, plug-in hybrid electric vehicles; FCEVs, fuel cell electric vehicles.

| **Passenger Cars Types** | **Fuel Economy (Mkm/PJ)** | | | |
| --- | --- | --- | --- | --- |
|  | **Lidköping** | **Skara** | **Grästorp** | **Gothenburg** |
| Gasoline ICEVs | 467 | 467 | 484.6 | 460.3 |
| Diesel ICEVs | 547.8 | 547.8 | 562 | 542.3 |
| HVO100 ICEVs | 547.8 | 547.8 | 562 | 542.3 |
| Ethanol ICEVs | 471.7 | 471.7 | 489.5 | 464.9 |
| Biogas ICEVs | 430.8 | 430.8 | 447.1 | 424.6 |
| Small BEVs | 1,733.2 | 1,733.2 | 1,653.9 | 1,767.1 |
| Average BEVs | 1,397.3 | 1,397.3 | 1,325.7 | 1,428.2 |
| Gasoline HEVs | 567.5 | 567.5 | 588.9 | 559.4 |
| Diesel HEVs | 669.8 | 669.8 | 686.3 | 663.4 |
| PHEVs | 991.6 | 991.6 | 993.5 | 990.8 |
| FCEVs | 828.5 | 828.5 | 859.8 | 816.6 |

The development of the existing stock of private cars over the modeled time horizon was assumed by calculating the residual stock (i.e., the probability of a car not retiring at a specific age). The residual stock was calculated according to a Weibull distribution following the same metrics (i.e., scale and shape) as used by the ON-TIMES energy system model open source^16^, Held et al.^37^, and Morfeld et al.^38^(see Figure S3). For new investments in passenger cars, a constraint on a lifetime of 17 years was implemented ^16^.


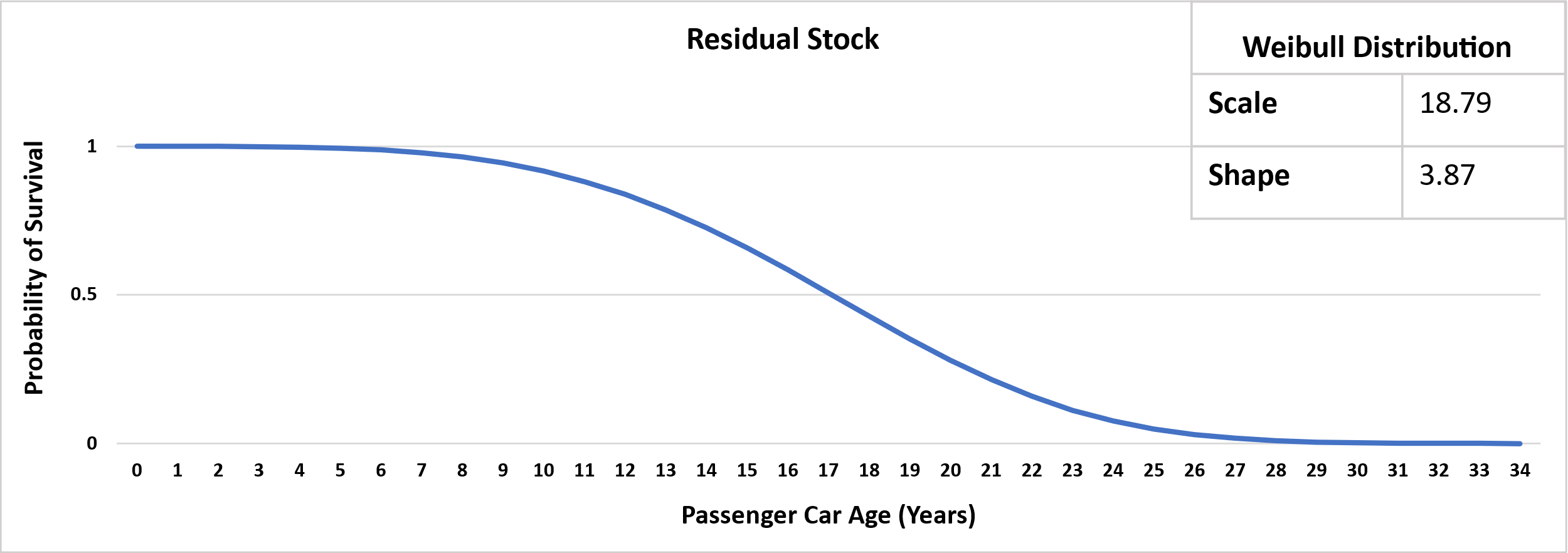


Figure S3 – Residual stock of the existing passenger cars calculated through a Weibull distribution. The stock curves show the probability of a given existing passenger car at a certain age not retiring. The scale and shape parameters result from an average of three different sources^16,37,38^. The residual stock was calculated based on the national registrations of the past five years^39^.

As investment cost, this model applied the same costs for all ICEVs and future decreasing EVs costs, as assumed by Helgeson et al.^40^. Small BEVs were not considered by the previous authors and so, this model applied the investment cost for these vehicles as suggested by Hagos et al.^4^. The operation and maintenance costs adopted the values suggested by Hagos et al.^4^. A description of both operation and maintenance as well as investment costs is presented in Table S10.

Table S10 – Economic input data used for passenger cars^4,40^. ICEVs, internal combustion engine vehicles; BEVs, battery electric vehicles; HEVs, hybrid electric vehicles; PHEVs, plug-in hybrid electric vehicles; FCEVs, fuel cell electric vehicles; NA, non-applicable.

| **Passenger Cars Types** | **Investment Cost (€)** | | | **Operation & Maintenance Cost (€/km)** |
| --- | --- | --- | --- | --- |
|  | **2019** | **2030** | **2050** |  |
| Gasoline ICEVs | 22,573 | | | 0.027 |
| Diesel ICEVs | 24,373 | | | 0.032 |
| HVO100 ICEVs | 24,373 | | | 0.032 |
| Ethanol ICEVs | 22,573 | | | 0.027 |
| Biogas ICEVs | 24,631 | | | 0.027 |
| Small BEVs | 28,900 | 24,373 | 23,500 | 0.021 |
| Average BEVs | 31,042 | 25,790 | 24,646 | 0.021 |
| Gasoline HEVs | 26,535 | 26,164 | 25,420 | 0.027 |
| Diesel HEVs | 27,156 | 26,784 | 26,288 | 0.032 |
| PHEVs | 30,125 | 26,829 | 25,371 | 0.027 |
| FCEVs | NA | 25,700 | 23,700 | 0.025 |
| **References** | ^4,40^ | | | ^4^ |

**Buses**

As previously mentioned, bus passenger transport was described at a regional level of Västra Götaland. According to the registrations of buses in 2019, directly retrieved from Trafikanalys 2019 statistics^12^, the existing stock was disaggregated into seven different engine types. As with passenger cars, these registrations do not clarify how many diesel buses are running on diesel or HVO100, to which this study applied F3’s assumption^41^ that 72% of the whole diesel bus fleet runs on HVO100. The same engines are fed to the model as an investment option, with the possibility of investing in FCEV buses starting in 2030.

Vehicle occupancy and annual mileage resulted from combining different data sources^16,17^ and are presented in Table S3. The fuel economy for the different bus engines was retrieved from Hagos et al.^4^. Yet, bus fuel economy data on gasoline ICEVs, HVO100 ICEVs, and ethanol (ED95) ICEVs were missing in the referred database, and thus, these values were estimated by applying the same difference between the equivalent passenger engine cars and diesel ICEVs (i.e., fuel economy of these three bus engines was calculated by multiplying the diesel ICEVs bus fuel economy by the ratio between fuel economy of gasoline ICEVs, HVO100 ICEVs, and ethanol ICEVs passenger cars and diesel ICEVs passenger cars). Both existing stock and future investments were modeled by assuming a lifetime of eight years, as applied by the ON-TIMES energy system model open source^16^.

Economic data – investment and operation and maintenance cost – integrated the same values as used by Hagos et al^4^, as aligned with JRC-EU-TIMES database^34^ (a database often used and referred to in existing TIMES literature). For the investment cost and operation and maintenance cost of ICEVs running on gasoline, HVO100, and ethanol (ED95), the same metric, as used to determine fuel economy (i.e., these costs were estimated by applying the same difference between passenger cars running on these fuels and diesel ICEVs to diesel ICEVs buses costs), was applied.

Buses’ techno-economic data is thoroughly presented Table S11.

Table S11 - Techno-economic input data used for buses^4,12,16,31^. ICEVs, internal combustion engine vehicles; BEVs, battery electric vehicles; HEVs, hybrid electric vehicles; PHEVs, plug-in hybrid electric vehicles; FCEVs, fuel cell electric vehicles; NA, Non-applicable.

| **Buses Types** | **Existing Stock (Number of Vehicles** | **Fuel Economy (Mkm/PJ)** | **Lifetime**  **(Years)** | **Investment Cost (€)** | | | **Operation & Maintenance Cost (€/km)** |
| --- | --- | --- | --- | --- | --- | --- | --- |
|  |  |  |  | **2019** | **2030** | **2050** |  |
| Gasoline ICEVs | 4 | 93 | 8 | 193,355 | | | 0.580 |
| Diesel ICEVs | 601 | 118.1 |  | 208,050 | | | 0.624 |
| HVO100 ICEVs | 1547 | 118.1 |  | 208,050 | | | 0.624 |
| Ethanol ICEVs | 0 | 92.9 |  | 193,355 | | | 0.580 |
| Biogas ICEVs | 378 | 91.5 |  | 234,733 | | | 0.704 |
| BEVs | 74 | 416. 7 |  | 242,415 | 233,020 | 224,463 | 0.364 |
| Diesel HEVs | 79 | 157.6 |  | 237,180 | 233,960 | 230,750 | 0.712 |
| FCEVs | NA | 256.4 |  | NA | 270,620 | 266,900 | 0.406 |
| **References** | ^12,41^ | ^4^ | ^16^ | ^4^ | | | |

**Freight Transport**

Freight transport was modeled at the Västra Götaland regional level, and disaggregated into medium-duty (i.e., gross truck weight < 3.5 ton) and heavy-duty (i.e., gross truck weight >= 3.5 ton). According to the truck registrations presented in Trafikanalys 2019 statistics^12^, medium-duty trucks included eight different engines, and heavy-duty trucks included seven different engines. As previously assumed for passenger cars and buses, this study applied the same ratio according to which 30% of diesel trucks run on HVO100^31^. For the medium-duty trucks, as previously assumed for passenger cars, it was assumed a normal distribution between the existing gasoline and diesel HEVs. For both medium-duty and heavy-duty trucks, this study modeled the same engines as existing in the base year stock as future truck investment options and that PHEVs and FCEVs can be chosen from 2020 and 2030, respectively. No new investments in gasoline ICEVs were considered for heavy trucks.

Freight vehicle occupancy and annual mileage adopted the same values as the ON-TIMES energy system model open source^16^ and are presented in detail in Table S3. The fuel economy for the different truck engines was retrieved from Hagos et al.^4^. Similar to the situation observed with buses, there was an absence of fuel economy data pertaining to gasoline, HVO100, and ethanol ICEVs. The fuel economy of these engines was estimated by using the same metric as applied for buses (i.e., the fuel economy of these three engines was determined by taking the diesel truck's fuel economy and multiplying it by the ratio of the fuel economy of gasoline, HVO100, and ethanol passenger cars to that of diesel passenger cars). As applied by the ON-TIMES energy system model open source^16^, a lifetime of 20 years and seven years was modeled for both existing stock and future investments of medium and heavy trucks, respectively.

Trucks’ economic data —investment, operation, and maintenance costs—are adopted from Hagos et al. projections^4^. The investment cost and operation and maintenance cost of ICEVs running on gasoline, HVO100, and ethanol were calculated using the same metric as used in fuel economy (i.e., these costs were estimated by applying the same difference between passenger cars running on these fuels and diesel ICEVs to diesel ICEV truck costs).

The techno-economic data implemented for medium-duty and heavy-duty trucks is described in Table S12.

Table S12 - Techno-economic input data used for tucks^4,12,16,31^. ICEVs, internal combustion engine vehicles; BEVs, battery electric vehicles; HEVs, hybrid electric vehicles; PHEVs, plug-in hybrid electric vehicles, FCEVs, fuel cell electric vehicles; M, medium truck (i.e., gross truck weight < 3.5 ton); H, heavy truck (i.e., gross truck weight >= 3.5 ton); NA, non-applicable.

| **Trucks Types** | | **Existing Stock (Number of Vehicles** | **Fuel Economy (Mkm/PJ)** | **Lifetime**  **(Years)** | | **Investment Cost (€)** | | | | | **Operation & Maintenance Cost (€/km)** |
| --- | --- | --- | --- | --- | --- | --- | --- | --- | --- | --- | --- |
|  |  |  |  |  |  | **2019** | **2030** | | **2050** | |  |
|  |  |  |  | **L** | **H** |  |  |  |  |  |  |
| Gasoline ICEVs | **M** | 6,440 | 336.7 | 20 | 6 | 34,200 | | | | | 0.236 |
|  | **H** | 161 | 118. 5 |  |  | NA | | | | | 0.284 |
| Diesel ICEVs | **M** | 53,877 | 363.6 |  |  | 35,400 | | | | | 0.245 |
|  | **H** | 9,489 | 150.5 |  |  | 174,214 | | | | | 0.306 |
| HVO100 ICEVs | **M** | 23,090 | 363.6 |  |  | 35,400 | | | | | 0.245 |
|  | **H** | 4,067 | 150.5 |  |  | 174,214 | | | | | 0.306 |
| Ethanol ICEVs | **M** | 205 | 336. 4 |  |  | 34,200 | | | | | 0.236 |
|  | **H** | 155 | 118.4 |  |  | NA | | | | | 0.284 |
| Biogas ICEVs | **M** | 1,629 | 295.9 |  |  | 38,940 | | | | | 0.269 |
|  | **H** | 3 | 122.5 |  |  | 198,445 | | | | | 0.336 |
| BEVs | **M** | 576 | 1123.6 |  |  | 49,200 | | 44,760 | | 39,120 | 0.170 |
|  | **H** | 5 | 447.4 |  |  | 194,030 | | 188,540 | | 183,810 | 0.171 |
| Gasoline HEVs | **M** | 3 | 540. 6 |  |  | 38,990 | | 38,710 | | 38,710 | 0.269 |
|  | **H** | NA | |  |  | NA | | | | | |
| Diesel HEVs | **M** | 3 | 485.4 |  |  | 40,360 | | 39,810 | | 39,260 | 0.279 |
|  | **H** | 4 | 201 |  |  | 205,655 | | 201,550 | | 199,485 | 0.348 |
| PHEVs | **M** | NA | 555. 6 |  |  | 44,400 | | 41,640 | | 39,120 | 0.307 |
|  | **H** |  | 229.9 |  |  | 226,265 | | 223,200 | | 220,130 | 0.383 |
| FCEVs | **M** | NA | 787.4 |  |  | NA | | 45,298 | | 44,677 | 0.136 |
|  | **H** |  | 326.8 |  |  | NA | | 226,608 | | 223,410 | 0.171 |
| **References** | | ^12,31^ | ^4^ | ^16^ | | ^4^ | | | | | |

1. **Emissions Mitigation Target**

In the light of the Paris Agreement, Sweden has established a national climate target of achieving net zero domestic GHG by 2045^10^. To offset the corresponding emission, some regions, like Västra Götaland, decided on an intermediate domestic target that encourages each municipality to become fossil-fuel independent (i.e., fossil-fuel independence is described at the municipal level as “the emissions of GHGs must be reduced by 80 percent from the 2010 level by the year 2030”^9^). Both targets were captured in this model, by setting one intermediate and one long-term model constraint. The intermediate constraint reflected the municipal commitment to becoming fossil fuel-independent by 2030. The long-term constraint set the climate neutrality national target to be met by 2045. Important to note that as previously mentioned, only CO_2_ emissions were considered in this study.

No data was found to specify the total CO_2_ emissions from road transport in 2019, both at the regional level of Västra Götaland and the municipal level of the participating municipalities. Therefore, this model determined the emissions baseline (CO_2_ emissions in 2019) by making a separate model run for a single year using 2019 input data. The resulting emissions were set as the baseline and thereafter both intermediate and long-term climate targets were applied. From the base year, CO_2_ emissions were constrained to be reduced annually, and linearly, until reaching the given targets. Both intermediate and long-term CO_2_ reduction targets are summarized in Table S13.

Table S13 – CO2 emission reduction targets according to both intermediate and long-term climate targets. NA, non-applicable.

| **Road Transport CO_2_ Emission Reduction Targets** | | | |
| --- | --- | --- | --- |
| **Year** | **Municipality Fossil Fuel Independence** | **CO_2_ Emissions** | **Model Assumption** |
| Base Year (2019) | No | 0% | CO_2_ emissions reduction targets are calculated relative to the emissions modeled in 2019. |
| Municipal Climate Target (2030) | Yes | 80% | **Intermediate Target:** The model constrains the total CO_2_ emissions to be reduced by 80%, compared to 2010. |
| National Climate Target (2045) | Yes | 100% | **Long-term Target:** The model constrains the total CO_2_ emission to be zero. |

1. **Calibration**

As explained by Loulou et al.^11^, model base year calibration is one of the most important stages of TIMES model development for two main reasons. First, the model base year corresponds to a past timeframe, restricting the model’s flexibility by user-defined model variables, constraints, and parameters set based on statistical and historical data. Second, developing a TIMES model is a very heavy data process, merging different and independent data sources, compromising the representation of the energy system under study. Such a calibration can be done by, according to a historic year and corresponding official energy statistics, setting all model variables as fixed^42^. This study chose the years 2020 to 2022, and related road transport data, as a calibration metric. Accordingly, the model's (i) existing stock (i.e., vehicles new registration and retirement profiles)^43^; (ii) the annual mileage^44^; (iii) transport demand; (iv) total CO_2_ emissions^25^; (v) fossil fuel composition in terms of biofuel blended share^11^; and (vi) electricity (and consequently hydrogen produced via electrolysis) emission factor resulting from the existing power system^25^; were calibrated relative to official national statistics.

1. **Model Scenarios**

This study aimed to understand the potential impact of different socio-geographical contexts on road transport fuel choices. The assessment was done by testing different CO_2_ reduction scenarios on meeting both the municipality fossil fuel independence 2030 and zero CO_2_ emissions 2045 targets. Due to the scope of this study, a local energy system perspective was added to this study by modeling local road transport scenarios developed in collaboration with municipal officials, previously presented by de Oliveira Laurin et al.^45^. These local scenarios were implemented in the model as a sensitivity analysis tool that tested different local futures based on both the challenges and opportunities identified by local authorities when meeting road transport decarbonization goals.

***No Policy Scenario***

Based on today’s road transport context of the modeled municipalities and region as well as fuel composition and costs, this scenario runs over the whole model time horizon devoid of climate mitigation targets and related policies. The *No Policy Scenario* operates as a baseline and thus, is used as a comparison metric to the other scenarios.

***Climate Policy Scenario***

This scenario applies two CO_2_ emissions reduction targets as well as existing and projected to-be-implemented policies directly impacting road transport, at the European^46,47^, Swedish National^11,26,48–52^, and Västra Götalands’ Regional-Municipal^53^ levels. First, under this scenario, the model is constrained to meet the municipal fossil fuel independence target by 2030. Accordingly, passenger cars’ total CO_2_ emissions are required to be reduced by 80% compared to 2010. Second, according to the national climate goal, the model constrains that the total CO_2_ emissions, including both light-duty vehicles (passenger cars), buses as well as medium and heavy-duty trucks –, to be zero, by 2045.

***Local Scenarios***

A local perspective was added to the modeling exercise by including three different local scenarios. Due to their local dimension, these scenarios were integrated into this study to perform a sensitivity analysis on the potential local futures of the intervening municipalities. The local scenarios were identified in already published work by the first author, with the local pathways generation guidelines being identified and presented by de Oliveira Laurin et al.^45^. According to the authors, local road transport pathways were generated through the development of an iterative and co-development process of combining different phases of literature review and iterative discussions with researcher-municipal officials. As a context-dependent method, the authors generated and presented road transport decarbonization pathways targeting the local context of the same three non-urban municipalities as the ones modeled in this study. Yet, the pathways presented in^45^ were limited to a qualitative description, missing, thus, a quantitative validation, which this study is adding.

In this study, three local pathways are transcribed into three different modeling local scenarios. The formulation of local scenarios evolves a set of quantitative (modeling) assumptions. Such modeling assumptions are used better to capture the qualitative description of the given local scenarios, as presented by de Oliveira Laurin et al.^45^:

- ***Self-sufficiency Electricity Scenario:*** This scenario assumes that the passenger cars’ electricity demand, on an annual average basis, is met locally, by means of variable renewable energy sources (VRESs) – solar and wind. Such a modeling scenario limits the municipal electricity demand of passenger cars not to exceed the VRESs annual average local electricity production. Within this limit, electricity, as in the base case, is perceived as a fuel that can be consumed at the base case fuel cost. Yet, if the local existing electricity production limit is met, the model can invest in new VRESs. According to this scenario, modeling results will provide an assessment of fuel shift, meaning that the model will choose between (i) investing in local electricity production – VRESs –, in case needed to meet the local passenger car electricity demand, or (ii) shifting passenger cars’ electricity demand towards another fuel. In the case of investing in new local electricity production sites, the cost for electricity will be a model result.

**Specific input data and modeling assumptions:**

Techno-economic input data regarding existing VRESs as well as possible future investments are specifically considered in this scenario, as presented in Table S14. Important to note is that investment in energy storage is disregarded, yet the intermittency of VRESs is exogenously captured by assuming an annual availability, representing the capacity factor.

Table S14 - Techno-economic input data for the eligible electricity production technologies, according to the Self-sufficiency Electricity scenario. The municipalities' label colors match the scheme used in the map presented in Figure S1. The future maximum annual electricity generation accounts for the capacity factors of the VRESs power plants. Only wind onshore capacity is considered and no solar PV rooftop is considered. PV, Photovoltaic.

| **Technology** | **Municipality** | **Current Annual Electricity Generation (TJ)** | **Future Maximum Annual Electricity Generation (TJ)** | **Investment Cost (€/kW_el_)** | **Maintenance and Operation Cost** | | **Lifetime (yr)** | **Annual Availability (%)** |
| --- | --- | --- | --- | --- | --- | --- | --- | --- |
|  |  |  |  |  | **Fixed (€/kW_el_/yr)** | **Variable (€/kWh_el_)** |  |  |
| Wind | Lidköping | 324 | 2,816.5 | 1240 | 10 | 1.1 | 25 | 33 |
|  | Skara | 651.6 | 1623 |  |  |  |  |  |
|  | Grästorp | 3.6 | 1,032.4 |  |  |  |  |  |
|  | Gothenburg | 10.8 | 33 |  |  |  |  |  |
| Solar PV | Lidköping | 25.2 | 4419 | 60 | 10 | 1.1 | 25 | 14 |
|  | Skara | 10.8 | 1,680.2 |  |  |  |  |  |
|  | Grästorp | 7.2 | 1,759 |  |  |  |  |  |
|  | Gothenburg | 136.8 | 13.1 |  |  |  |  |  |
| **References** | | ^54^ | ^55^ | ^56^ | | | | ^57^ |

- ***Bio-locked Scenario***: In this study, the bio-locked modeling scenario limits the total biofuel road transport demand (passenger cars, buses, and trucks) to what is regionally produced. Within the Västra Götaland region, only biogas, and biodiesel (HVO100) were found to be regionally produced, meaning that ethanol (E85 and ED95) was disregarded as a fuel option in this scenario. Within the regional production capacity, biogas and biodiesel are consumed at the base case fuel cost. Accordingly, the model will test the regional cross-road transport segments of the existing biogas and biodiesel among the three road transport segments. Such an allocation is expected to highlight the monetary willingness that different road transport segments have to utilize these biofuels.

**Specific input data and modeling assumptions:**

Figure S4 and Table S15 presents the total annual average regional production of both biogas and biodiesel considered specifically in this scenario. These values represent the activity of biogas and biodiesel refineries currently existing in the considered region^58^.


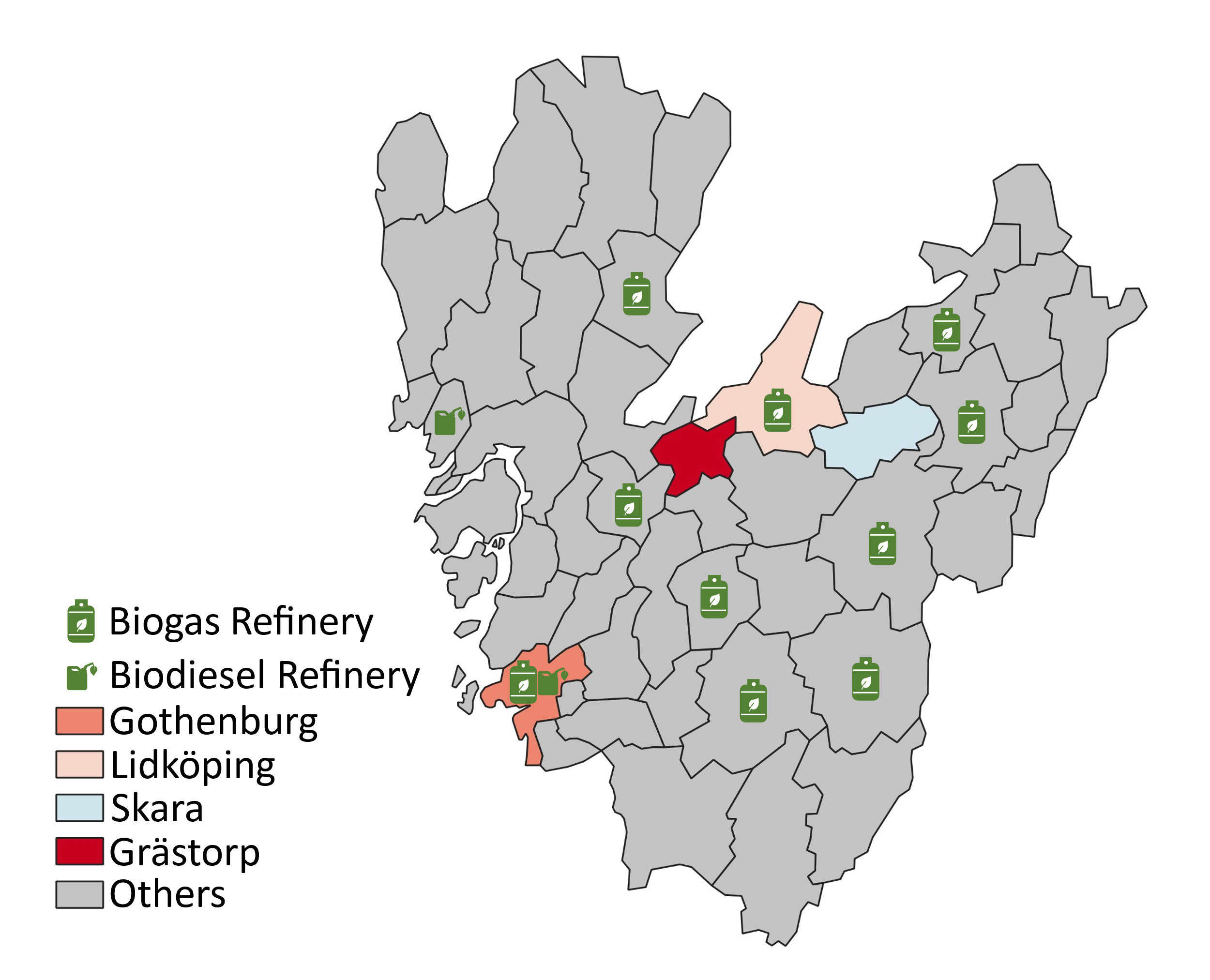


Figure S4- Map over Västra Götaland region with the corresponding location of current existing regional biogas and biodiesel (HVO100) refineries. The municipalities covered in this study are identified with orange, red, pink, and light blue. The municipalities not considered in this study are colored in grey. Regional map was generated for the specific purpose of this study, using the Quantum Geographic Information System (QGIS) 3.36.2^3^. Data retrieved from^58^.

Table S15 - Current annual average biogas and biodiesel (HVO100) production, both from municipal and aggregated regional perspectives. The municipalities' label colors match the scheme used in the map presented in Figure S1. Data retrieved from^58^.

| **Refinery Type** | **Municipality** | **Current Annual Average Regional Fuel Production (TJ)** | **Total Annual Average Regional Fuel Production (TJ)** |
| --- | --- | --- | --- |
| Biogas | Lidköping | 108 | 1,126.8 |
|  | Gothenburg | 446.4 |  |
|  | Borås | 108 |  |
|  | Falköping | 28.8 |  |
|  | Mariestad | 108 |  |
|  | Mellerud | 108 |  |
|  | Skövde | 108 |  |
|  | Trollhättan | 108 |  |
|  | Ulricehamn | 3.6 |  |
|  | Vårgårda | 108 |  |
| Biodiesel (HVO100) | Gothenburg | 44,496 | 82,296.0 |
|  | Lysekil | 37,800 |  |
| **References** | | ^58^ | |

- ***Flexible Public Transport*:** This scenario is modeled by exogenously incorporating a modal shift from passenger cars to public transport. Such a shift is perceived in the model by reducing the demand for passenger car transport in the same proportion as the demand for bus passenger transport increases. Modeling this modal shift, due to the decrease in total passenger car transport demand, is expected to provide flexibility to the development of the existing stock (i.e., the existing private car stock will require to drive less, and thus, it might be able to fit during for longer time the model fossil fuel independence and CO_2_ emissions constraints).

**Specific input data and modeling assumptions:**

Under this scenario, the regional plans for public transport demand are included, as increasing the annual bus passenger demand by 0.7 % until 2040 and by 0.5% until 2050^59^. Accordingly, the annual car passenger car is reduced in the same proportion as the bus transport demand increases.

Overall, Figure S5 summarizes the framework of transcribing the local pathways qualitatively described by de Oliveira Laurin et al.^45^ into modeling quantitative local road transport scenarios. This process called for a rationale attribution of different model assumptions in the format of model parameters, decision variables, and constraints. According to the local model set of assumptions, extra outputs were added to the model agenda results. Similarly, the different local scenarios were modeled to target different road transport segments.


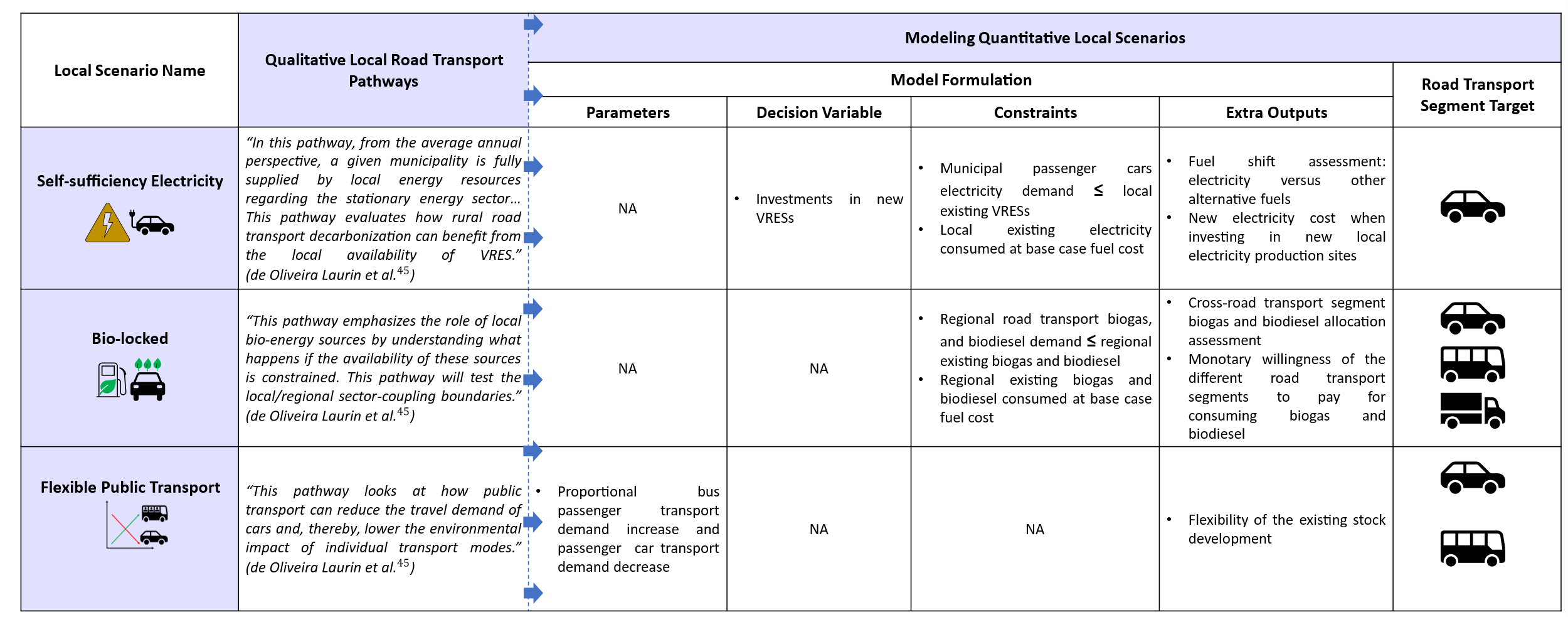


Figure S5 – Transcription of qualitative local road transport pathways, previously presented by de Oliveira Laurin et al.^45^ in modeling quantitative local road transport scenarios. VRESs, variable renewable energy sources; HVO, hydrotreated vegetable oil; NA, non-applicable.

1. **Additional Results**

The stock for buses, medium, and heavy-duty vehicles was aggregated in the *No Policy* and *Climate Policy Scenarios*. Figure S6-S17 present the stock separated for buses, medium, and heavy-duty vehicles


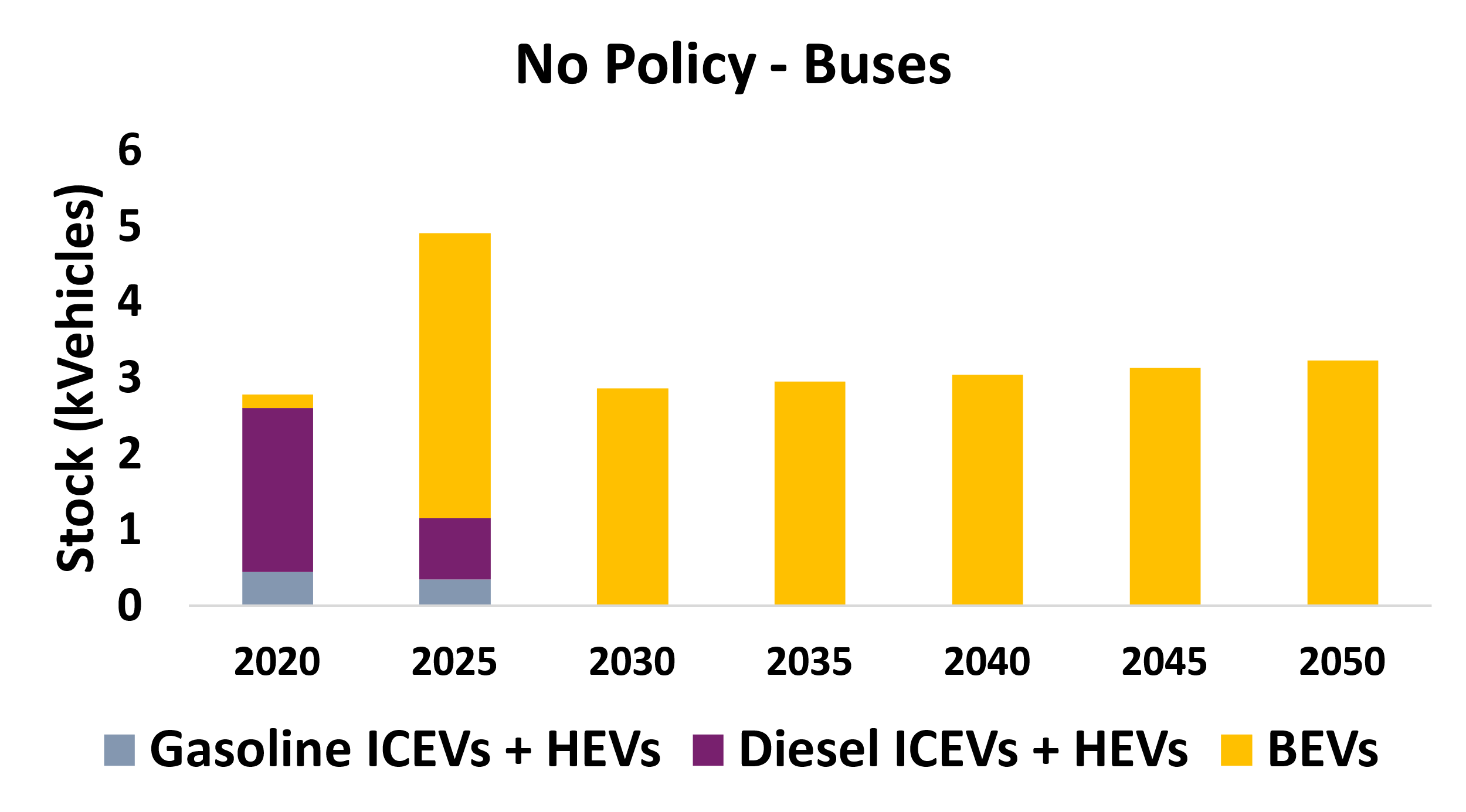


Figure S6 – Bus fleet mix presented in stock of thousand (k) vehicles for the No Policy Scenario. ICEVs, internal combustion engine vehicles; HEVs, hybrid electricity vehicles; BEVs, battery electric vehicles.


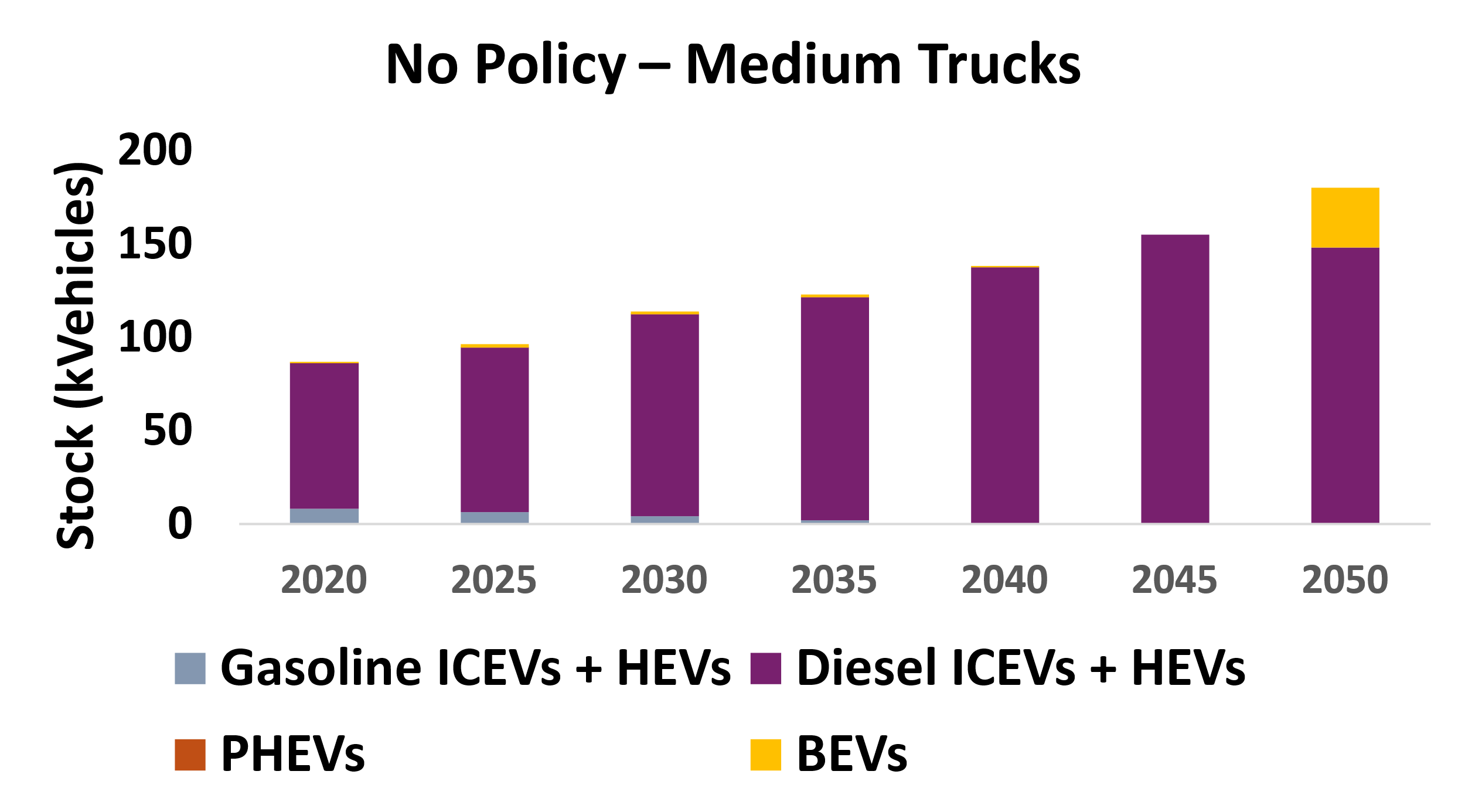


Figure S7 – Medium-duty trucks fleet mix presented in stock of thousand (k) vehicles for the No Policy Scenario. ICEVs, internal combustion engine vehicles; HEVs, hybrid electricity vehicles; PHEVs, plug-in hybrid vehicles; BEVs, battery electric vehicles.


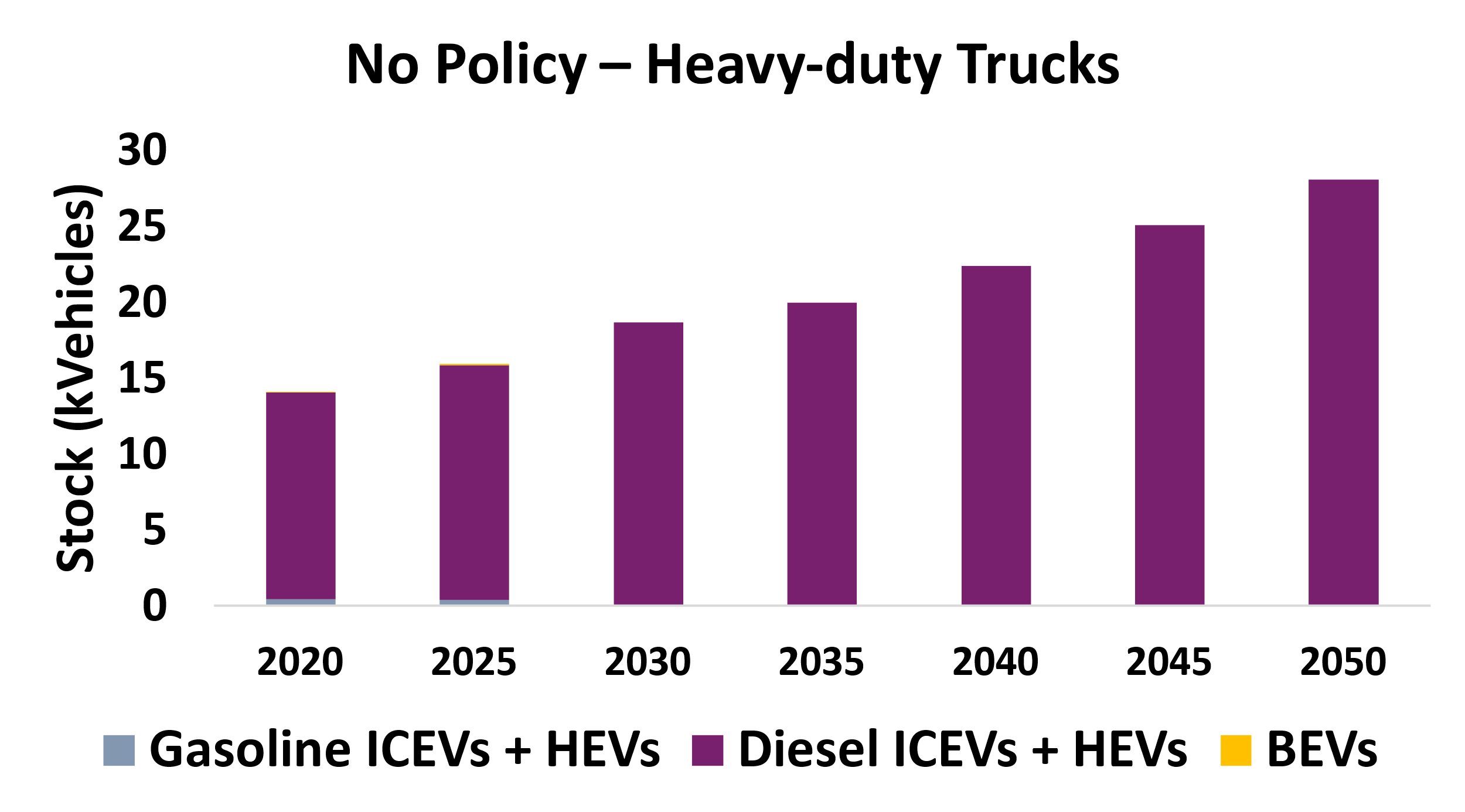


Figure S8 – Heavy-duty trucks fleet mix presented in stock of thousand (k) vehicles for the No Policy Scenario. ICEVs, internal combustion engine vehicles; HEVs, hybrid electricity vehicles; BEVs, battery electric vehicles.


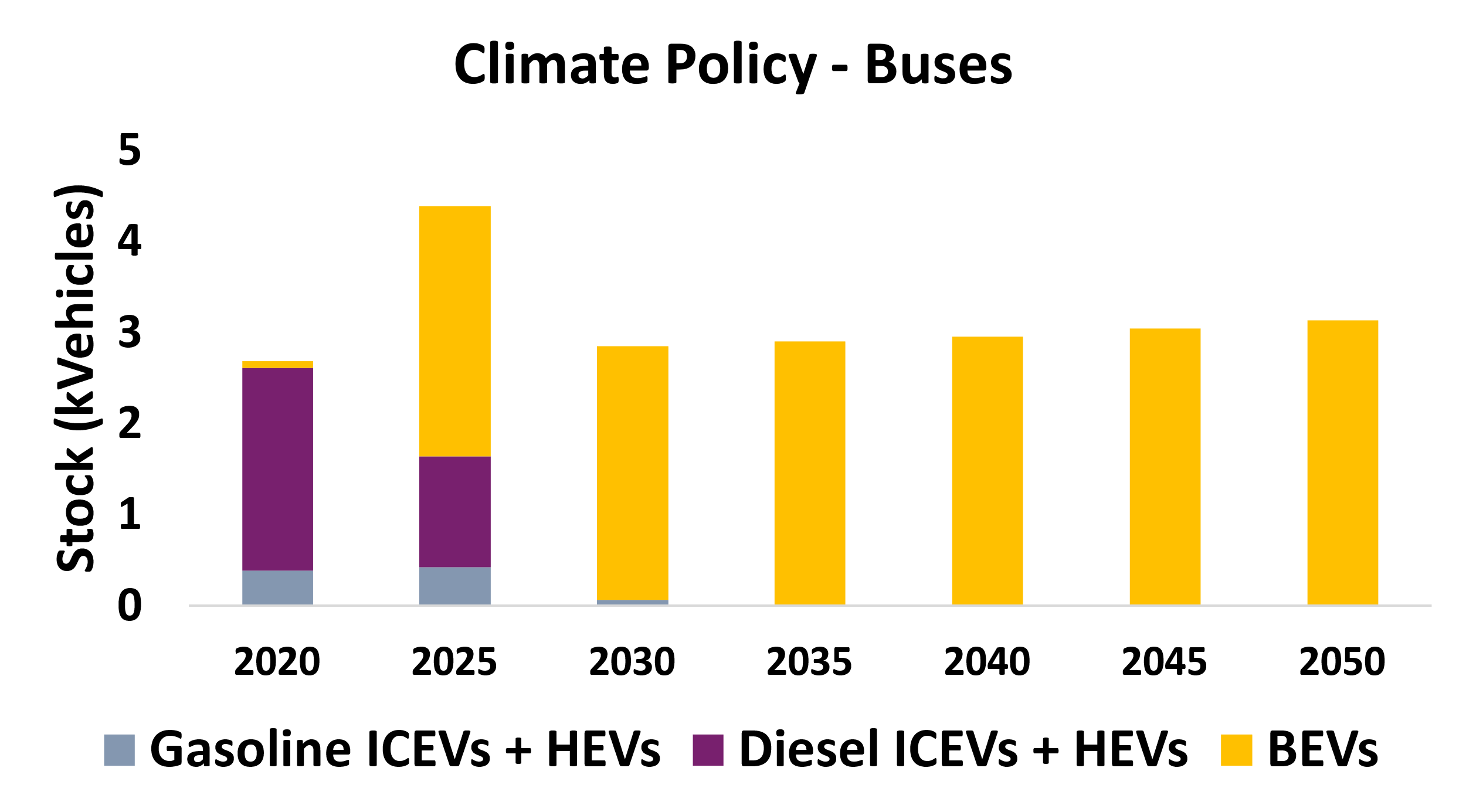


Figure S9 – Bus fleet mix presented in stock of thousand (k) vehicles for the Climate Policy Scenario. ICEVs, internal combustion engine vehicles; HEVs, hybrid electricity vehicles; BEVs, battery electric vehicles.


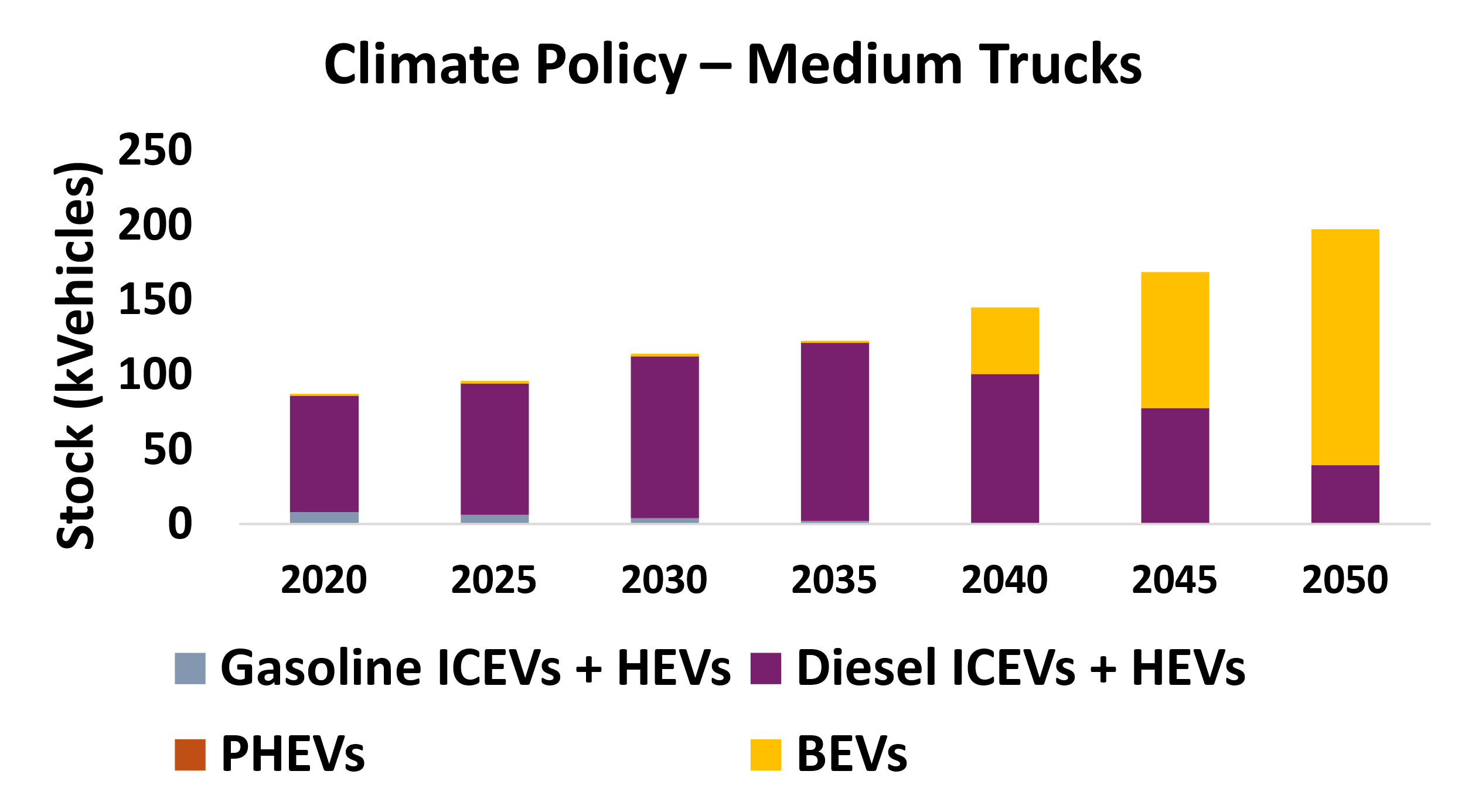


Figure S10 – Medium-duty trucks fleet mix presented in stock of thousand (k) vehicles for the Climate Policy Scenario. ICEVs, internal combustion engine vehicles; HEVs, hybrid electricity vehicles; PHEVs, plug-in hybrid vehicles; BEVs, battery electric vehicles.


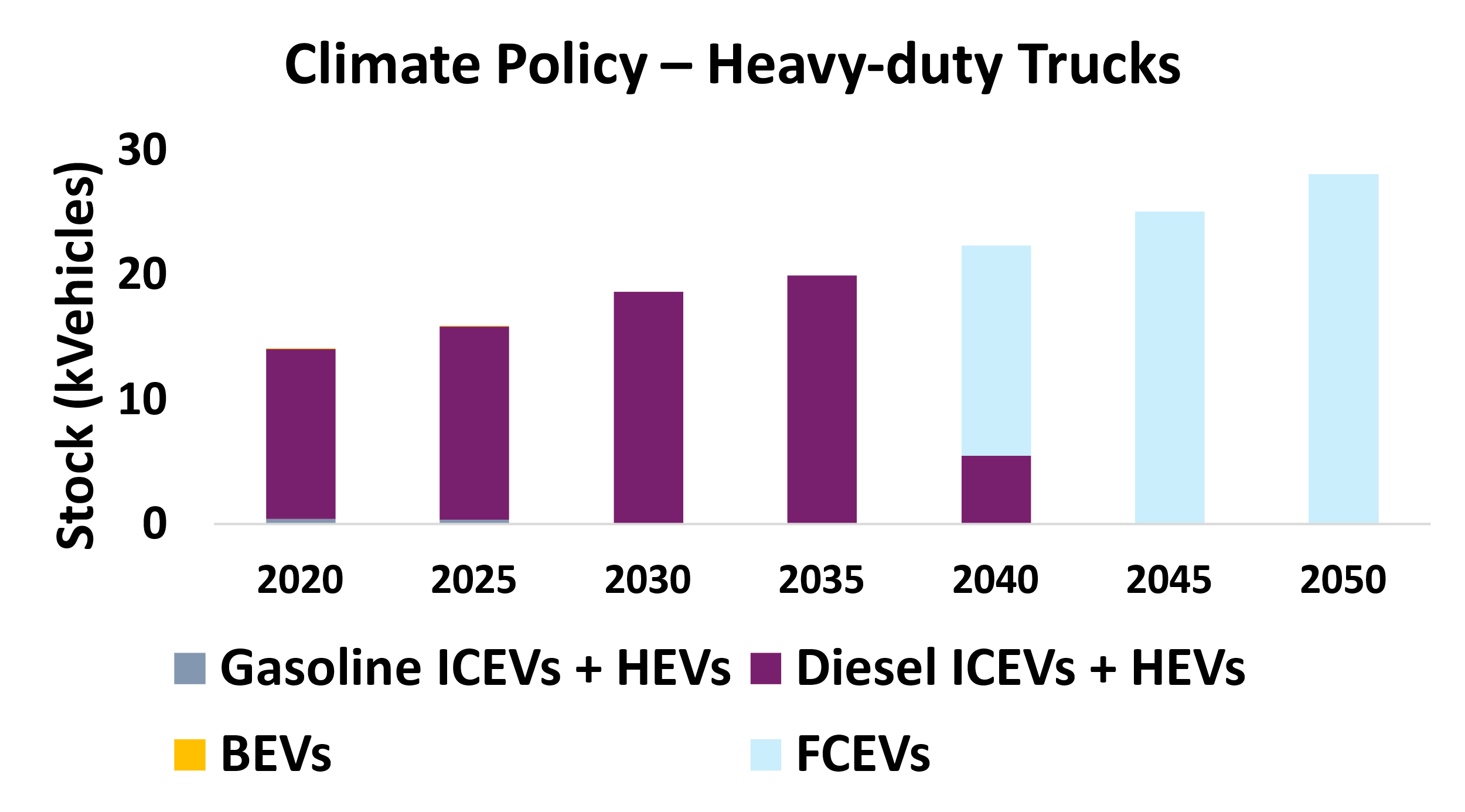


Figure S11 – Heavy-duty trucks fleet mix presented in stock of thousand (k) vehicles for the Climate Policy Scenario. ICEVs, internal combustion engine vehicles; HEVs, hybrid electricity vehicles; BEVs, battery electric vehicles; FCEVs, fuel cell electric vehicles.


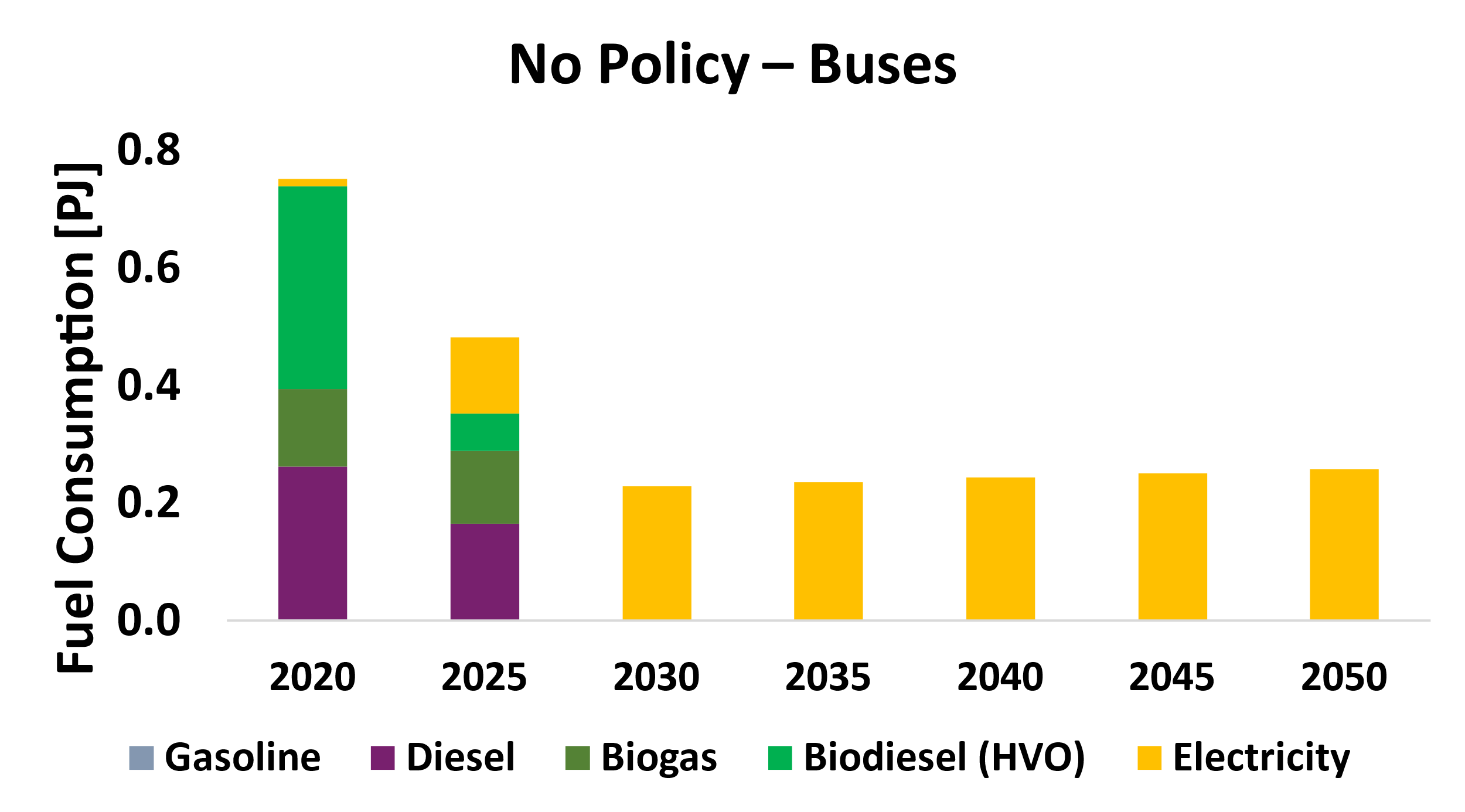


Figure S12 – Bus fuel consumption for the No Policy Scenario. HVO, hydrotreated vegetable oil.


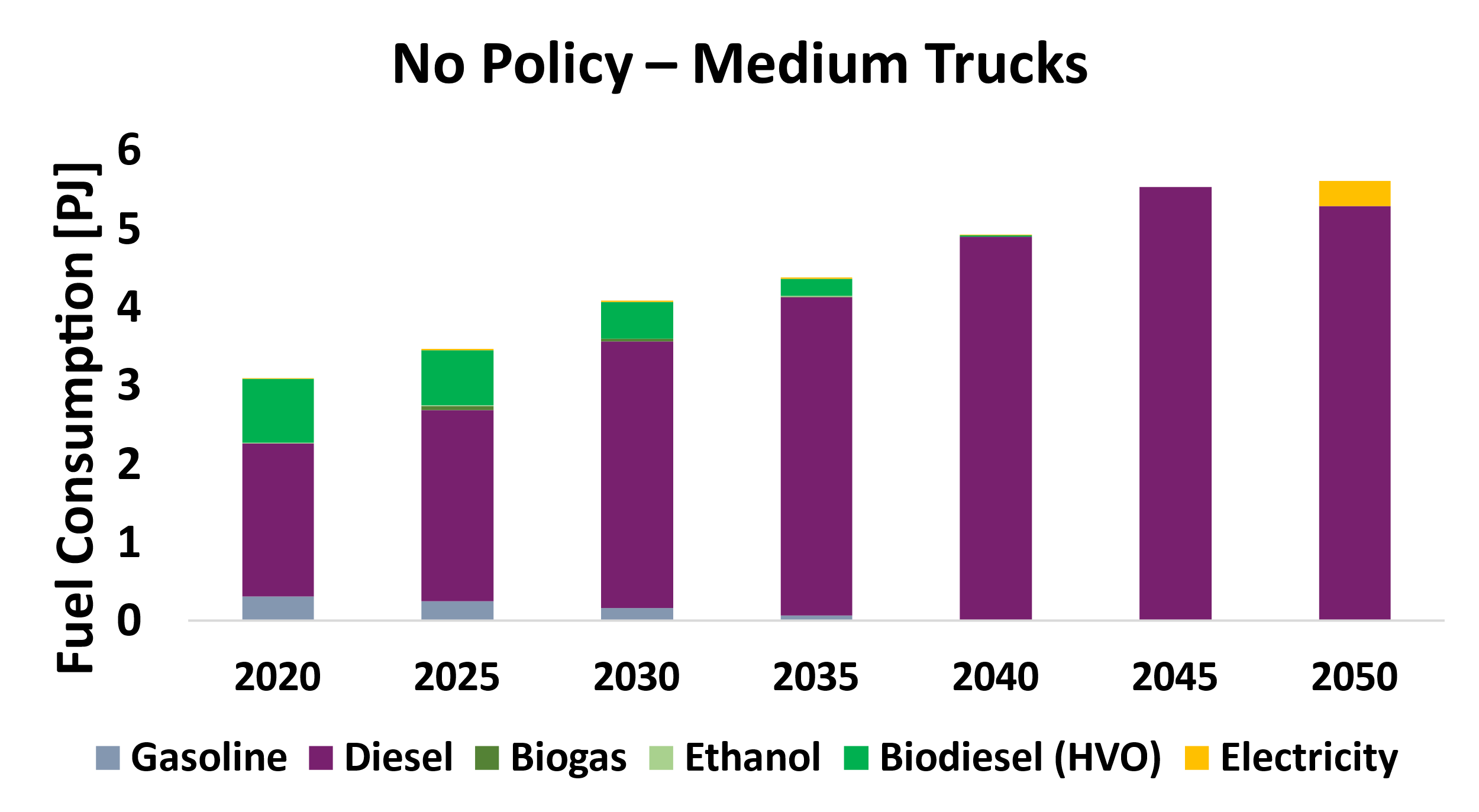


Figure S13 – Medium-duty trucks fuel consumption for the No Policy Scenario. The ethanol considered for light-duty vehicles is E85 and for medium and heavy-duty vehicles ED95. HVO, hydrotreated vegetable oil.


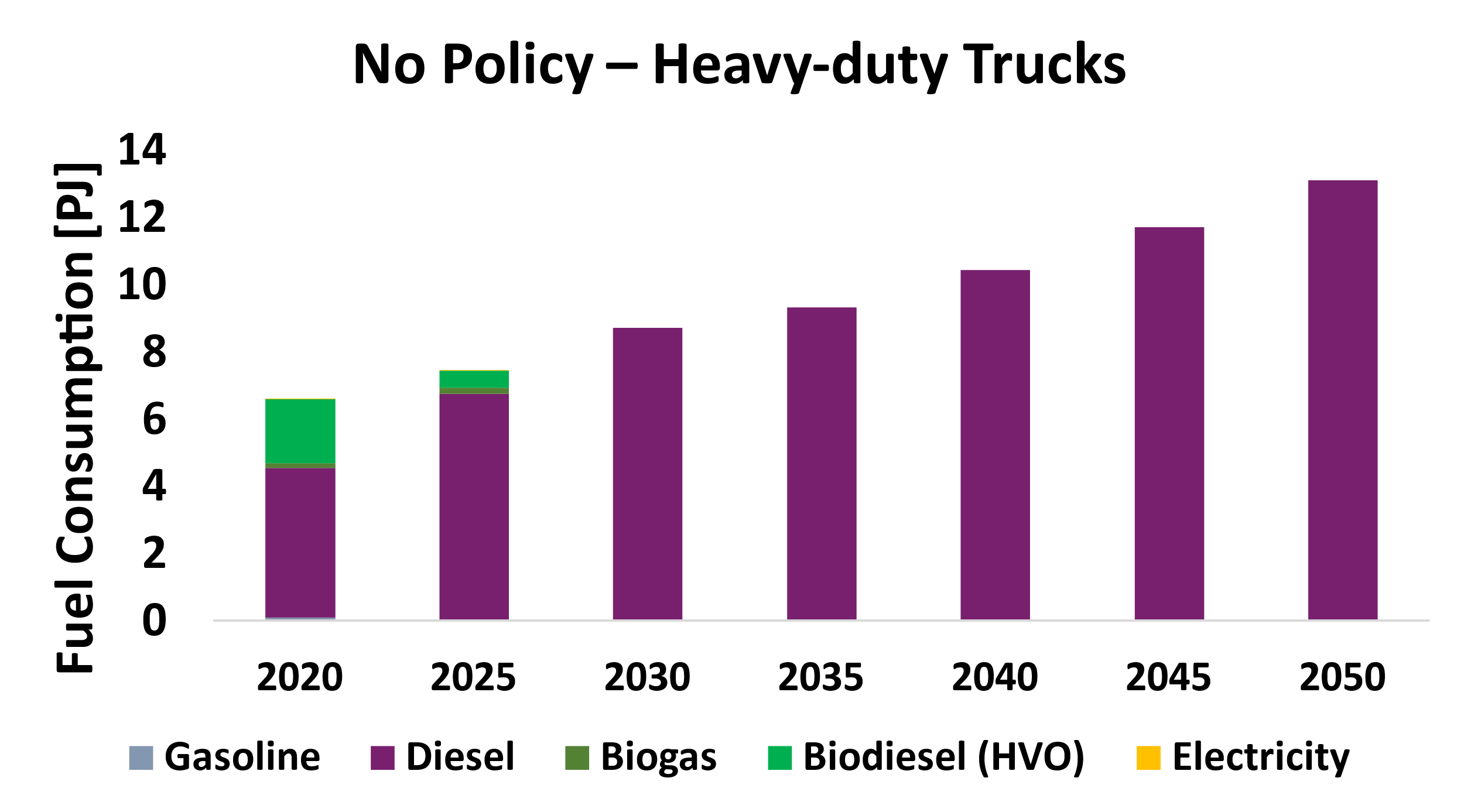


Figure S14 – Heavy-duty trucks fuel consumption for the No Policy Scenario. HVO, hydrotreated vegetable oil.


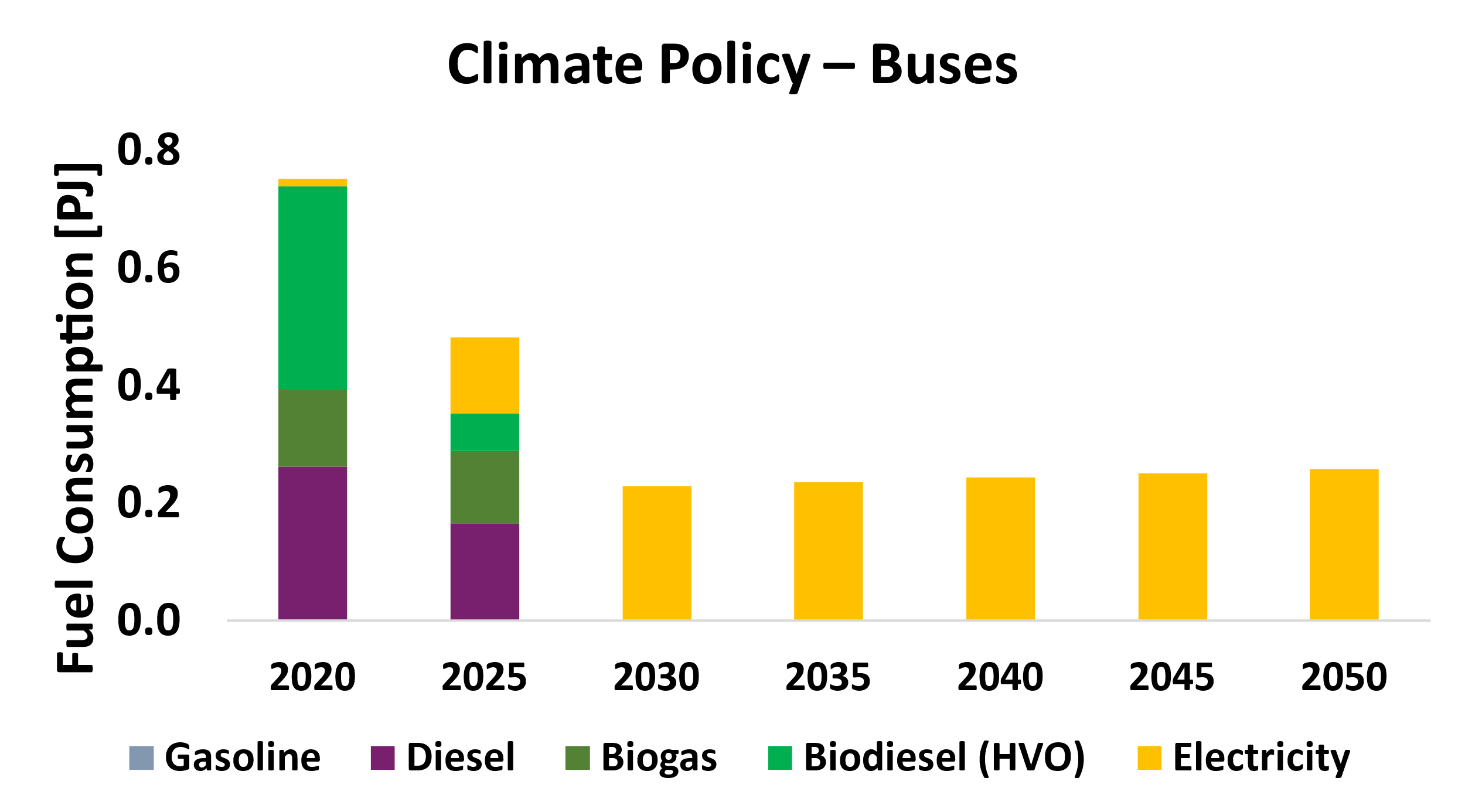


Figure S15 – Buses fuel consumption for the Climate Policy Scenario. HVO, hydrotreated vegetable oil.


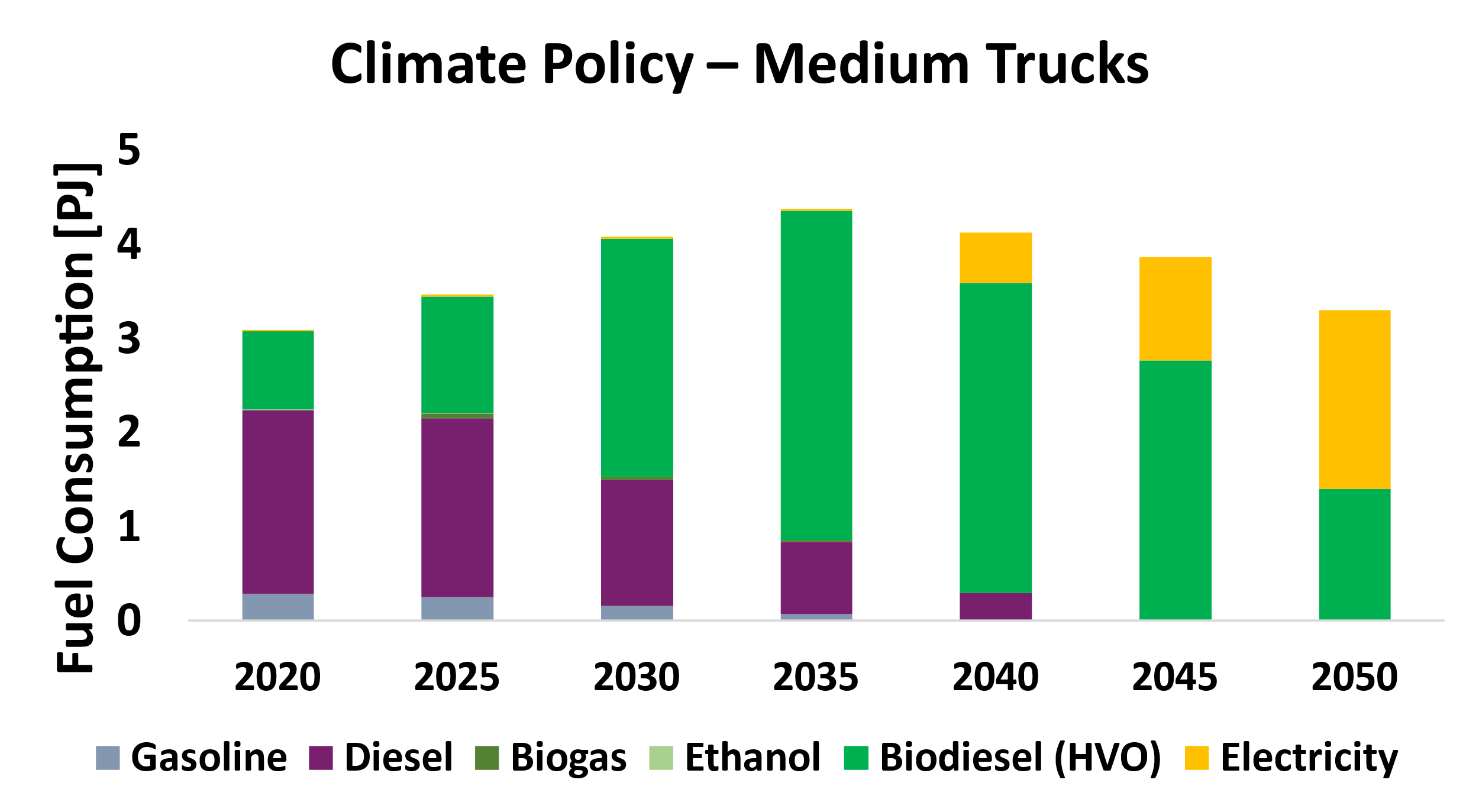


Figure S16 – Medium-duty trucks fuel consumption for the Climate Policy Scenario. The ethanol considered for light-duty vehicles is E85 and for medium and heavy-duty vehicles ED95. HVO, hydrotreated vegetable oil.


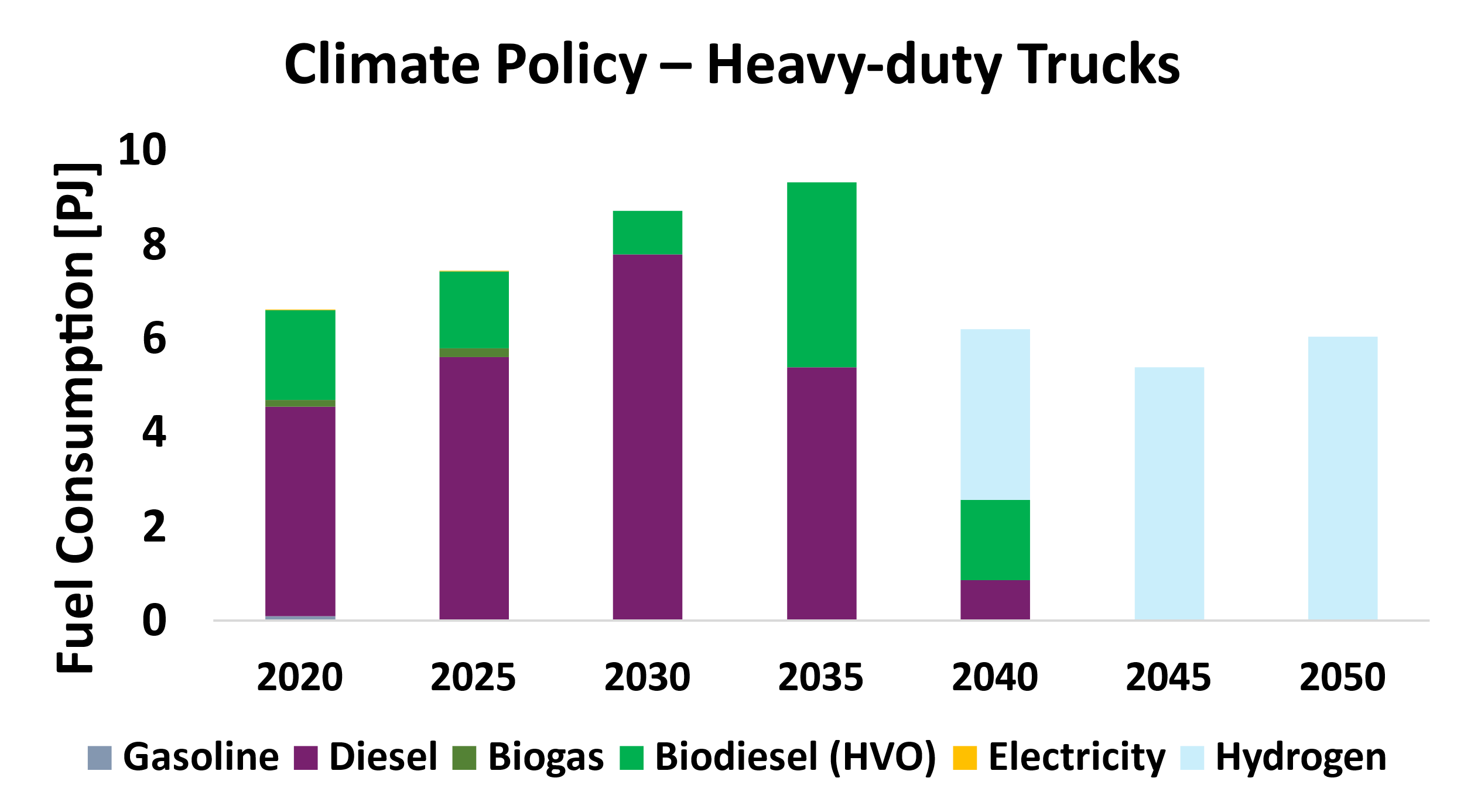


Figure S17 – Heavy-duty trucks fuel consumption for the Climate Policy Scenario. HVO, hydrotreated vegetable oil.

The focus of the modeled local scenarios was the passenger car fleet. Yet, for both *Bio-locked* and *Flexible Public Transport Scenarios*, both buses as well as medium and heavy-duty trucks were further included in the modeling exercise.

Figure S18 depicts the stock for these vehicles under the two abovementioned local scenarios, while Figure S19, for the same vehicles and local scenarios, illustrates the fuel mix.

It is important to note that testing the *Flexible Public Transport Scenario* resulted in an increase in the bus stock. Nevertheless, as the total number of buses, compared to trucks, is considerably low, results (as are presented aggregating both buses as well as medium and heavy-duty trucks) did not change considerably when compared to the *Climate Policy Scenario* presented in the main manuscript.

*
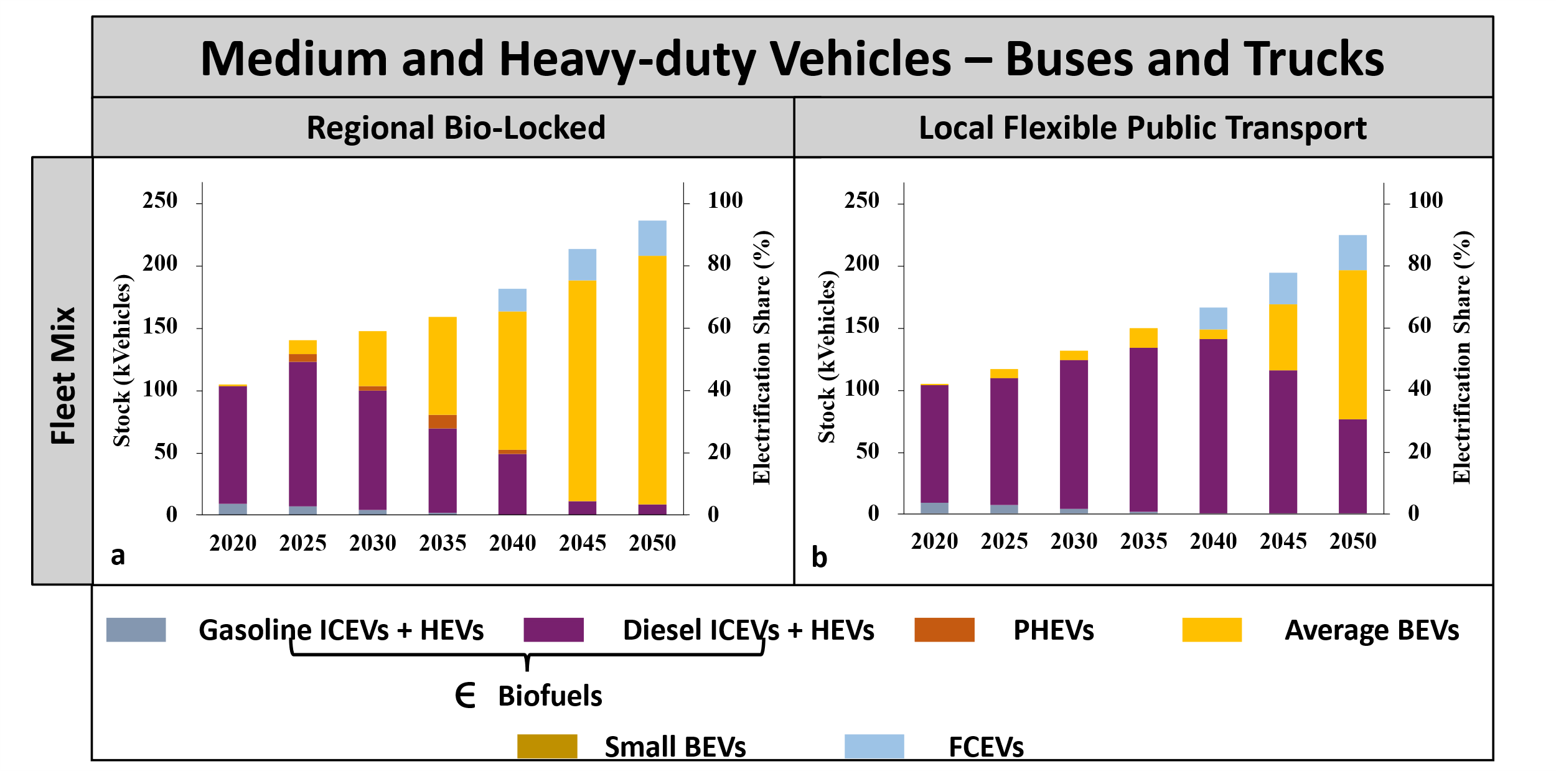
Figure S18 - Fleet mix presented in stock of thousand (k) vehicles (left axes). The black and blue curves show the electrification share (right axes). ICEVs, internal combustion engine vehicles; HEVs, hybrid electricity vehicles; PHEVs, plug-in hybrid vehicles; BEVs, battery electric vehicles; FCEVs, fuel cell electric vehicles.*


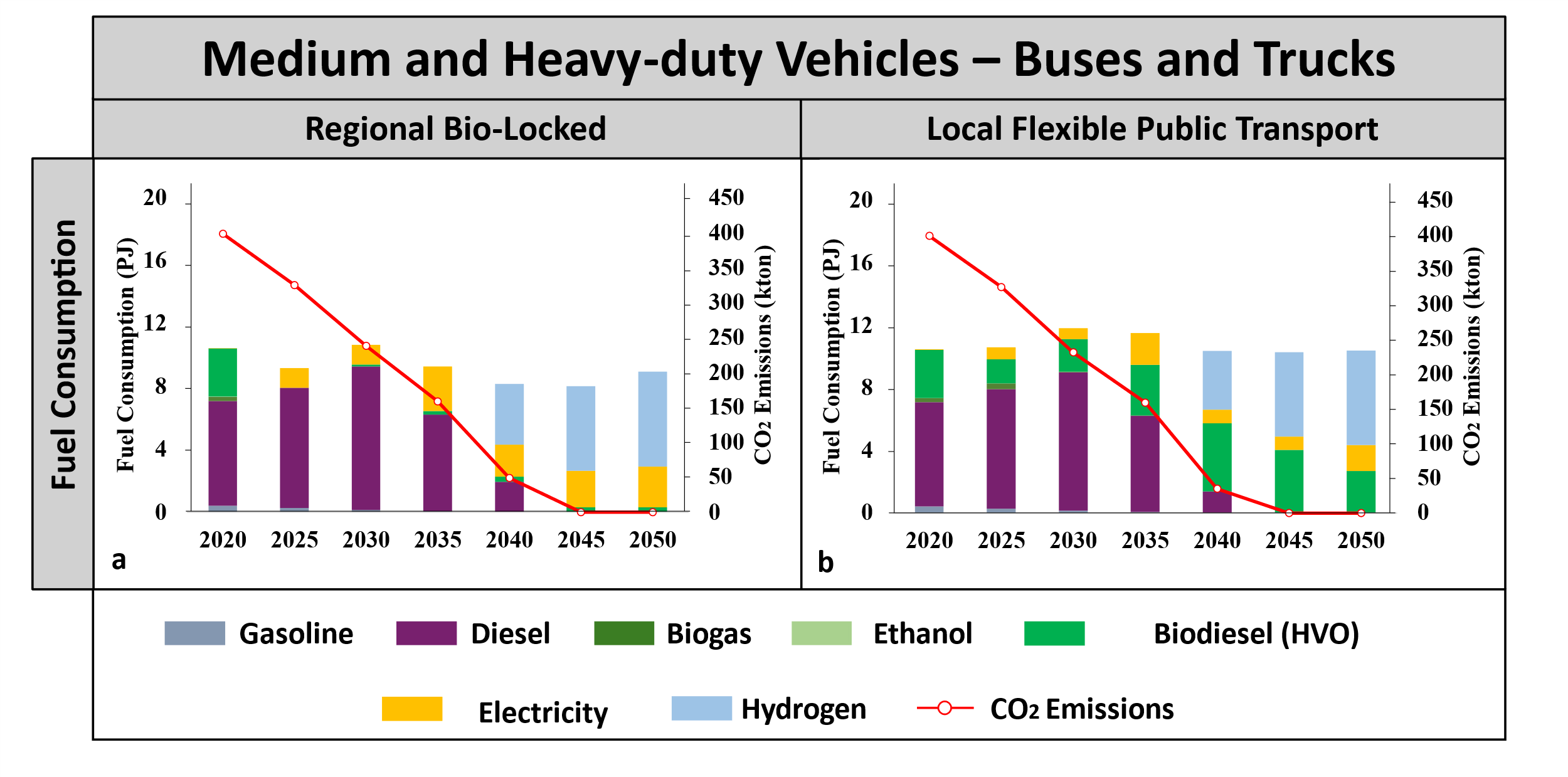
*Figure S19 - Fuel consumption (left axes) and resulting CO2 emissions (right axes). The ethanol considered for light-duty vehicles is E85 and for medium and heavy-duty vehicles ED95. HVO, hydrotreated vegetable oil.*

**Supplementary Information References**

1. Åhlvik, K. & Gillingsjö, G. Kommungruppsindelning 2017 Omarbetning av Sveriges Kommuner och Landsting Kommungruppsindelning. https://webbutik.skr.se/bilder/artiklar/pdf/7585-455-7.pdf (2016).
2. Sveriges kommuner & Landsting. Classification of Swedish Municipalities 2017. https://skr.se/en/skr/tjanster/kommunerochregioner/faktakommunerochregioner/kommungruppsindelning.2051.html (2017)
3. Quantum Geographic Information System. https://www.qgis.org/.
4. Hagos, D. A. & Ahlgren, E. O. Exploring cost-effective transitions to fossil independent transportation in the future energy system of Denmark. Appl Energy 261, 114389, DOI: https://doi.org/10.1016/j.apenergy.2019.114389 (2020).
5. Bunch, D. S., Ramea, K., Yeh, S. & Yang, C. Incorporating behavioral effects from vehicle choice models into bottom-up energy sector models. Institute of Transportation Studies, University of California, Davis, Davis, California (2015).
6. Krook Riekkola, A. et al. Outline of the City-level modelling framework. (2018).
7. Balyk, O. et al. TIM: modelling pathways to meet Ireland’s long-term energy system challenges with the TIMES-Ireland Model (v1. 0). Geosci Model Dev 15, 4991–5019, DOI: https://doi.org/10.5194/gmd-15-4991-2022 (2022).
8. Krook Riekkola, A. National Energy System Modelling for Supporting Energy and Climate Policy Decision-making: The Case of Sweden. (2015).
9. Götalandsregionen, V. & Länsstyrelsen. Klimat 2030 - Västra Götaland ställer om. Preprint at https://klimat2030.se/ (2017).
10. Naturvårdsverket. Sveriges klimatmål och klimatpolitiska ramverk. https://www.naturvardsverket.se/amnesomraden/klimatomstallningen/sveriges-klimatarbete/sveriges-klimatmal-och-klimatpolitiska-ramverk/ (2017) .
11. Sverige Riskdag. Lag (2017:1201) om reduktion av växthusgasutsläpp från vissa fossila drivmedel. https://rkrattsbaser.gov.se/sfst?bet=2017:1201 (2017).
12. Trafikanalys. Vehicles in counties and municipalities 2019. https://www.trafa.se/vagtrafik/fordon/ (2019).
13. Trafikverket. Analysmetod Och Samhällsekonomiska Kalkylvärden För Transportsektorn: ASEK 7.1. Trafikverket, Borlänge, Sweden https://bransch.trafikverket.se/contentassets/4b1c1005597d47bda386d81dd3444b24/2023/asek-7.1-hela-rapporten-2023-09-20.pdf (2023).
14. EEA. Are we moving in the right direction? Indicators on transport and environmental integration in the EU: TERM 2000 - Occupancy rates. <https://www.eea.europa.eu/publications/ENVISSUENo12/page029.html> (2000).
15. Tozluoğlu, Ç. et al. A synthetic population of Sweden: datasets of agents, households, and activity-travel patterns. Data Brief 48, 109209, DOI: https://doi.org/10.1016/j.dib.2023.109209 (2023).
16. Nordic Energy Research. The entire ON-TIMES energy system model is available on GitHub. https://github.com/NordicEnergyResearch/NCES2020 (2021).
17. Trafikanalys. The Swedish national travel survey 2019. https://www.trafa.se/kommunikationsvanor/RVU-Sverige/ (2019).
18. Länsstyrelserna. Car mileage until 2022. https://rus.se/statistik-och-indikatorer/korstrackor/. (2023)
19. Andersen, F. M., Jacobsen, H. K. & Gunkel, P. A. Hourly charging profiles for electric vehicles and their effect on the aggregated consumption profile in Denmark. International Journal of Electrical Power & Energy Systems 130, 106900 DOI: <https://doi.org/10.1016/j.ijepes.2021.106900> (2021).
20. Lajevardi, S. M., Axsen, J. & Crawford, C. Simulating competition among heavy-duty zero-emissions vehicles under different infrastructure conditions. Transp Res D Transp Environ 106, 103254 DOI: https://doi.org/10.1016/j.trd.2022.103254 (2022).
21. Aryanpur, V. & Rogan, F. Decarbonising road freight transport: The role of zero-emission trucks and intangible costs. Sci Rep 14, 2113 DOI: <https://doi.org/10.1038/s41598-024-52682-4> (2024).
22. Trafikverket. Prognos För Persontrafiken 2040. https://bransch.trafikverket.se/contentassets/7e1063efbcfd4b34a4591b0d4e00f855/2023/rapport-prognos-for-persontrafik-trafikverkets-basprognos-2023-04-01.pdf (2023).
23. Forsberg, J. & Krook Riekkola, A. Recoupling climate change and air quality: Exploring low-emission options in urban transportation using the times-city model. Energies (Basel) 14, 3220, DOI: https://doi.org/10.3390/en14113220 (2021).
24. Energy Analyses. Fuel Costs – Production, Distribution and Infrastructure Costs Used in the Economic Analysis in Grøn Roadmap 2030. https://ea-energianalyse.dk/wp-content/uploads/2020/02/1459_fuel_costs_production_distribution_infrastructure.pdf (2015).
25. Nowtricity. Current emissions in Sweden. https://www.nowtricity.com/country/sweden/ (2023).
26. Naturvardsverket. Sweden’s Climate Act and Climate Policy Framework. https://www.naturvardsverket.se/en/international/swedish-environmental-work/swedens-climate-act-and-climate-policy-framework/#:~:text=In%202017%2C%20Sweden%20adopted%20a,by%202045%20at%20the%20latest.
27. Juhrich, K. CO2 Emission Factors for Fossil Fuels. German Environment Agency (2016).
28. Miljöfordon.se. Etanolbil. https://www.miljofordon.se/bilar/etanolbil/ (2022).
29. Hallberg, L. et al. Well-to-wheel LCI data for fossil and renewable fuels on the Swedish market. Report from an f3 project. The Swedish Knowledge Centre for Renewable Transportation Fuels. Sweden. https://f3centre.se/app/uploads/f3-Report-2013-29_LCI-Database_141215.pdf (2013).
30. Miljöfordon.se. Miljöpåverkan. https://www.miljofordon.se/bilar/miljoepaaverkan/ (2023).
31. International Energy Agency Bioenergy. Country Report: Implementation of Bioenergy in Sweden – 2021 Update. https://www.ieabioenergy.com/wp-content/uploads/2021/11/CountryReport2021_Sweden_final.pdf (2021).
32. Safarian, S. Environmental and energy impacts of battery electric and conventional vehicles: A study in Sweden under recycling scenarios. Fuel Communications 14, 100083, DOI: https://doi.org/10.1016/j.jfueco.2022.100083 (2023).
33. Borlaug, B. et al. Estimating region-specific fuel economy in the United States from real-world driving cycles. Transp Res D Transp Environ 86, 102448, DOI: https://doi.org/10.1016/j.trd.2020.102448 (2020).
34. Sofia, G. D. A. C. S. et al. The JRC-EU-TIMES model-assessing the long-term role of the set plan energy technologies. https://publications.jrc.ec.europa.eu/repository/handle/JRC85804 (2013).
35. Huss, A. & Weingerl, P. JEC Tank-To-Wheels Report v5: Passenger Cars. https://publications.jrc.ec.europa.eu/repository/handle/JRC117560 (2020).
36. Börjesson, P. et al. Methane as vehicle fuel–a well to wheel analysis (METDRIV). Report 6, f3. https://f3centre.se/app/uploads/f3_2016-06_borjesson-et-al_final_170111-1.pdf (2016).
37. Held, M., Rosat, N., Georges, G., Pengg, H. & Boulouchos, K. Lifespans of passenger cars in Europe: empirical modelling of fleet turnover dynamics. European Transport Research Review 13, 1–13, DOI: https://doi.org/10.1186/s12544-020-00464-0 (2021).
38. Morfeldt, J. & Johansson, D. J. A. Impacts of shared mobility on vehicle lifetimes and on the carbon footprint of electric vehicles. Nat Commun 13, 6400, DOI: https://doi.org/10.1038/s41467-022-33666-2 (2022).
39. Statistics Sweden. Vehicles in use by region and type of vehicles. Year 2002 - 2022. https://www.scb.se/en/finding-statistics/statistics-by-subject-area/transport-and-communications/road-traffic/vehicles/ (2023).
40. Helgeson, B. & Peter, J. The role of electricity in decarbonizing European road transport–Development and assessment of an integrated multi-sectoral model. Appl Energy 262, 114365, DOI: https://doi.org/10.1016/j.apenergy.2019.114365 (2020).
41. Xylia, M. & Silveira, S. Fuel options for public bus fleets in Sweden. https://www.diva-portal.org/smash/get/diva2:867716/FULLTEXT01.pdf (2015).
42. Loulou, R., Goldstein, G., Kanudia, A., Lehtila, A. & Remne, U. Documentation for the TIMES model: Part I 2016. https://iea-etsap.org/docs/Documentation_for_the_TIMES_Model-Part-I_July-2016.pdf (2016).
43. Trafikanalys. The Swedish national travel survey 2022. https://www.trafa.se/kommunikationsvanor/RVU-Sverige/ (2022).
44. Trafikanalys. Vehicles in counties and municipalities 2022. https://www.trafa.se/kommunikationsvanor/RVU-Sverige/ (2022).
45. de Oliveira Laurin, M., Selvakkumaran, S., O. Ahlgren, E. & Grahn, M. Are decarbonization strategies municipality-dependent? Generating rural road transport pathways through an iterative process in the Swedish landscape. Energy Research & Social Science, 114: 103570. https://doi.org/10.1016/j.erss.2024.103570 (2024).
46. European Parliament. EU ban on the sale of new petrol and diesel cars from 2035 explained. https://www.europarl.europa.eu/topics/en/article/20221019STO44572/eu-ban-on-sale-of-new-petrol-and-diesel-cars-from-2035-explained.
47. European Commission. ETS2 : buildings, road transport and additional sectors. https://climate.ec.europa.eu/eu-action/eu-emissions-trading-system-eu-ets/ets2-buildings-road-transport-and-additional-sectors_en.
48. Transportstyrelsen. Bonus malus-system för personbilar, lätta lastbilar och lätta bussar. https://www.transportstyrelsen.se/bonusmalus.
49. Johansson, B. Energibeskattningens utveckling i Sverige: En översiktlig historisk beskrivning. (2021).
50. Regeringskansliet. Sweden’s carbon tax. https://www.government.se/government-policy/swedens-carbon-tax/swedens-carbon-tax/#:~:text=Swedish%20carbon%20tax%20rates&text=The%20carbon%20tax%20was%20introduced,of%20SEK%2010.87%20per%20EUR). (2018).
51. Skatteverket. Skatt på bränsle. https://skatteverket.se/foretag/skatterochavdrag/punktskatter/energiskatter/skattpabransle.4.15532c7b1442f256bae5e56.html.
52. Regeringskansliet. Skattebefrielse för rena och höginblandade biodrivmedel till och med 2026. https://www.regeringen.se/pressmeddelanden/2022/12/skattebefrielse-for-rena-och-hoginblandade-biodrivmedel-till-och-med-2026/.
53. Götalandsregionen, V. & Länsstyrelsen. Klimat 2030 - Västra Götaland ställer om. Preprint at https://klimat2030.se/ (2017).
54. Länsstyrelserna. Kommunala sankey-diagram för Västra Götalands kommuner för år 2020. https://www.lansstyrelsen.se/download/18.186a2548185299da53ea0e3b/1675258209137/Kommunala-sankey-2020.pdf (2020).
55. Mattsson, N., Verendel, V., Hedenus, F. & Reichenberg, L. An autopilot for energy models–Automatic generation of renewable supply curves, hourly capacity factors and hourly synthetic electricity demand for arbitrary world regions. Energy Strategy Reviews 33, 100606, DOI: https://doi.org/10.1016/j.esr.2020.100606 (2021).
56. Johansson, V. & Göransson, L. Impacts of variation management on cost-optimal investments in wind power and solar photovoltaics. Renewable Energy Focus 32, 10–22, DOI: https://doi.org/10.1016/j.ref.2019.10.003 (2020).
57. Walter, V., Göransson, L., Taljegard, M., Öberg, S. & Odenberger, M. Low-cost hydrogen in the future European electricity system–Enabled by flexibility in time and space. Appl Energy 330, 120315, DOI: https://doi.org/10.1016/j.apenergy.2022.120315 (2023).
58. Fukushima, N., Vierth, I., Johansson, M. & Karlsson, R. Fossilt till bio: Klimatpåverkan av drivmedelsdistribution. https://www.diva-portal.org/smash/get/diva2:1818138/FULLTEXT01.pdf (2023).
59. Trafikverket. Prognos för persontrafiken 2040–Trafikverkets Basprognoser 2023-04-01. https://www.diva-portal.org/smash/get/diva2:1797550/FULLTEXT01.pdf (2023).
